# NAMPT‐Driven M2 Polarization of Tumor‐Associated Macrophages Leads to an Immunosuppressive Microenvironment in Colorectal Cancer

**DOI:** 10.1002/advs.202303177

**Published:** 2024-02-02

**Authors:** Sun Mi Hong, A‐Yeon Lee, Byeong‐Ju Kim, Jeong‐Eun Lee, Su‐Yeon Seon, Yu‐Jin Ha, Jestlin Tianthing Ng, Gyesoon Yoon, Su Bin Lim, Michael J. Morgan, Jong‐Ho Cha, Dakeun Lee, You‐Sun Kim

**Affiliations:** ^1^ Department of Biochemistry Ajou University School of Medicine 164 Worldcup‐ro, Yeongtong‐gu Suwon Gyeonggi‐do 16499 Republic of Korea; ^2^ Department of Biomedical Sciences Graduate School of Ajou University 164 Worldcup‐ro, Yeongtong‐gu Suwon Gyeonggi‐do 16499 Republic of Korea; ^3^ Department of Natural Sciences Northeastern State University Tahlequah OK 74464 USA; ^4^ Department of Biomedical Sciences College of Medicine Inha University Incheon 22212 South Korea; ^5^ Department of Biomedical Science and Engineering Graduate School Inha University Incheon 22212 South Korea; ^6^ Department of Pathology Ajou University School of Medicine 164 Worldcup‐ro, Yeongtong‐gu Suwon Gyeonggi‐do 16499 Republic of Korea

**Keywords:** CRC, HIF‐1α, M2‐like TAMs, NAMPT, STING

## Abstract

Nicotinamide phosphoribosyltransferase (NAMPT) is a metabolic enzyme with key roles in inflammation. Previous studies have examined the consequences of its upregulated expression in cancer cells themselves, but studies are limited with respect to its role in the other cells within the tumor microenvironment (TME) during colorectal cancer (CRC) progression. Using single‐cell RNA sequencing (scRNA‐seq) data, it is founded that *NAMPT* is highly expressed in *SPP1^+^* tumor‐associated macrophages (TAMs), a unique subset of TAMs associated with immunosuppressive activity. A *NAMPT^high^* gene signature in *SPP1^+^* TAMs correlated with worse prognostic outcomes in CRC patients. The effect of *Nampt* deletion in the myeloid compartment of mice during CRC development is explored. NAMPT deficiency in macrophages resulted in HIF‐1α destabilization, leading to reduction in M2‐like TAM polarization. NAMPT deficiency caused significant decreases in the efferocytosis activity of macrophages, which enhanced STING signaling and the induction of type I IFN‐response genes. Expression of these genes contributed to anti‐tumoral immunity via potentiation of cytotoxic T cell activity in the TME. Overall, these findings suggest that NAMPT‐initiated TAM‐specific genes can be useful in predicting poor CRC patient outcomes; strategies aimed at targeting NAMPT may provide a promising therapeutic approach for building an immunostimulatory TME in CRC progression.

## Graphical abstract

Nicotinamide phosphoribosyltransferase (NAMPT) is a metabolic enzyme with key role in inflammation. NAMPT plays a pivotal role in Tumor‐associated macrophages (TAMs) reprogramming by regulating the HIF‐1α/STAT3 and a reduction in STING activation. These molecular mechanisms imply that inhibition of NAMPT enzymatic activity or expression in tumor microenvironmental components is possibly a viable therapeutic strategy for colorectal cancer patients.

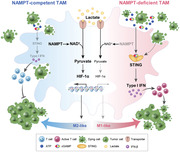

## 1 Introduction

Colorectal cancer (CRC) is the third most prevalent malignancy, and has a high mortality rate.^[^ ^1^ ^]^ CRC typically results from chromosomal instability with the development of mutations in specific oncogenes/tumor suppressor genes such as *TP53*, *KRAS*, and *APC*. However, the mutational landscape of tumor cells alone does not provide an accurate outcome of CRC progression.^[^ ^2^ ^]^ CRC has long been known as a cancer type highly associated with chronic inflammation;^[^ ^3^ ^]^ therefore it is important to understand how CRC cells communicate with the inflammatory microenvironment and the mechanism by which the tumor microenvironment (TME) influences CRC progression. Tumor cells interact with both immune and non‐immune cells to shape the complex cellular network of TME; they engage and drive the plasticity of TME cells including stroma cells, fibroblasts, endothelial cells, and immune cells.^[^ ^4^, ^5^ ^]^ It has been reported that exclusion of infiltrating immune cells from the TME is associated with a poor prognosis for CRC patients, while tumor‐associated macrophages (TAMs) localizing on the tumor margin prevent the infiltration of cytotoxic lymphocytes (CTL) into the tumor core.^[^ ^6^ ^]^ TAMs participate in multiple aspects of tumor immunity and can contribute to tumor progression.^[^ ^7^ ^]^ The “classically activated” M1 and “alternatively activated” M2 macrophage polarization terminologies have been used to describe the activation state of macrophages in vitro, however, in vivo TAMs exhibit more heterogenous and complex phenotypes.

Nicotinamide phosphoribosyltransferase (NAMPT) is the rate‐limiting enzyme in the nicotinamide adenine dinucleotide (NAD) salvage pathway, converting nicotinamide (NAM) into nicotinamide mononucleotide (NMN) which is a precursor of NAD.^[^ ^8^ ^]^ NAD is an essential cofactor that mediates various redox reactions to control cellular oxidative (catabolic) reactions; NAMPT is important for continuous replenishment of NAD levels in cells that exhibit high metabolic needs such as cancer cells and activated immune cells.^[^ ^9^ ^]^ Several reports have suggested that elevated NAMPT expression in various cancer cells promotes tumorigenesis.^[^ ^10^ ^]^ Targeting NAMPT to control tumorigenesis, however, is not simple: a greater understanding of roles of NAMPT in cancer cells and TME is necessary to develop possible therapeutics. Recently, single‐cell analysis of CRC has revealed that myeloid cells have a high degree of plasticity that is driven by signals within the TME and that the functional phenotypes of TAMs are associated with CRC progression and clinical outcome.^[^ ^11^, ^12^ ^]^ The role of NAMPT in TAMs and the TME has not been fully elucidated, particularly with regard to the pathogenesis, progression, and/or prognostic significance of CRC.

Here, we utilized mice with *Nampt* deletion in the myeloid compartment (*Nampt^f/f^ LysM cre^+/−^, Nampt* mKO) to explore the role of NAMPT in TAMs. NAMPT deficiency in macrophages reduced tumor growth in azoxymethane (AOM)/dextran sulfate sodium (DSS)‐induced colon cancer model and in xenograft model with an apparent decrease in “M2‐like” TAMs in the TME. NAMPT affects the phenotype of TAMs through several molecular mechanisms to contribute to immunosuppressive TME remodeling which promotes CRC progression.

## 2 Results

### 2.1 *NAMPT* Expression in Tumor‐Specific Macrophages is Associated with Colorectal Cancer Progression

**Figure 1:**
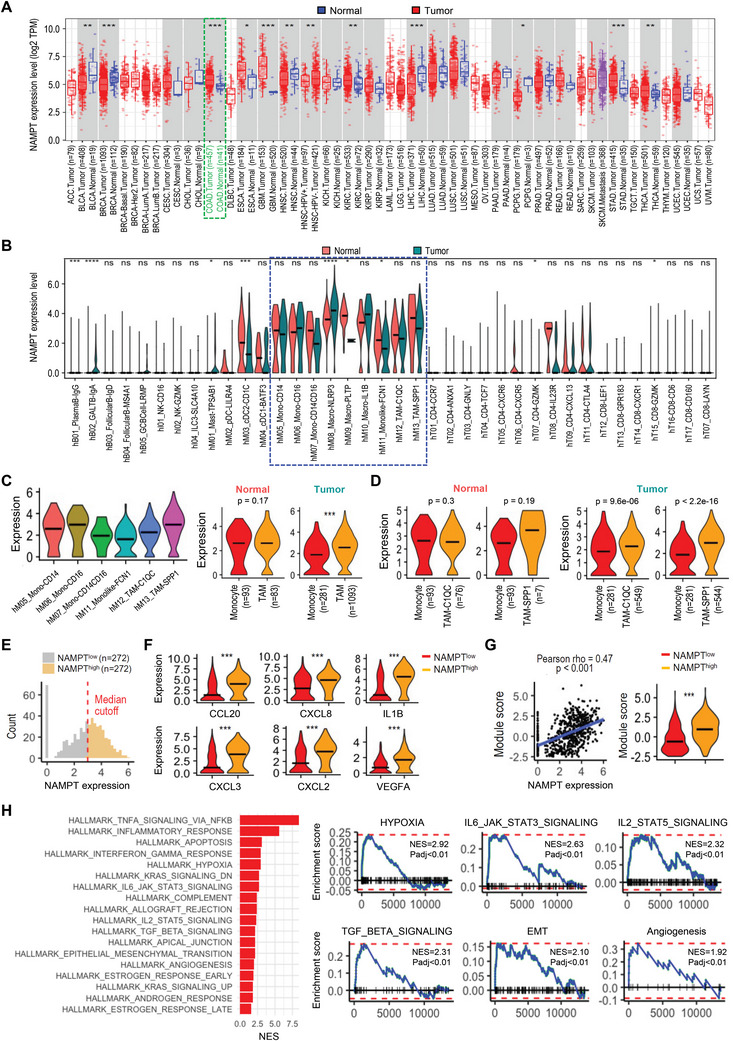
*NAMPT* is highly expressed in tumor‐specific macrophages associated with pro‐tumoral property of TAMs. A) The mRNA expression of *NAMPT* in tumor and adjacent normal tissues across all TCGA tumors is shown. The statistical significance was computed by the Wilcoxon test (^*^: *p*‐value <0.05; ^**^: *p*‐value <0.01; ^***^: *p*‐value <0.001). B) Relative expression of *NAMPT* mRNA in adjacent normal tissues and tumor tissues grouped by cell types is shown. The black line represents median expression. C) Relative expression of *NAMPT* mRNA is shown for tumor tissues grouped by selected myeloid cell clusters. The black line represents median expression. (left). Relative expression of *NAMPT* mRNA in monocytes (“hM05_Mono‐*CD14*”, “hM06_Mono‐*CD16*”, “hM07_Mono‐*CD14CD16*”, and “hM11_Monolike‐*FCN1*”) and TAMs (“hM12_TAM‐*C1QC*” and “hM13_TAM‐*SPP1*”) is shown for adjacent normal tissues (middle) and tumor tissues (right). n and p indicate the number of cells and t‐test p values (^*^<0.05, ^**^<0.01, ^***^<0.001), respectively. The black line represents median expression. D) Relative expression of *NAMPT* mRNA in monocytes (”hM05_Mono‐*CD14*”, “hM06_Mono‐*CD16*”, “hM07_Mono‐*CD14CD16*”, and “hM11_Monolike‐*FCN1*”) and TAMs (“hM12_TAM‐*C1QC*” or “hM13_TAM‐*SPP1*”) is shown for adjacent normal tissues (left) and tumor tissues (right). n and p indicate the number of cells and t‐test p values (^*^<0.05, ^**^<0.01, ^***^<0.001), respectively. The black line represents median expression. E) Density plot showing distribution of *NAMPT* expression in *NAMPT^high^* and *NAMPT^low^ SPP1^+^* TAMs. F) Relative expression of known TAM markers mediating M1/M2 polarization is shown for *SPP1^+^* TAMs. T‐test p values (^*^<0.05, ^**^<0.01, ^***^<0.001). The black line represents median expression. G) Correlation of *NAMPT* expression with module score of *CCL20, CXCL8, IL1B, CXCL3, CXCL2*, and *VEGFA* in *SPP1^+^* TAMs (left). Module scores of *NAMPT^high^* and *NAMPT^low^ SPP1^+^* TAMs are shown (right). T‐test p values (^*^<0.05, ^**^<0.01, ^***^<0.001) are shown. The black line represents median expression. H) Top enriched Hallmark gene sets in the *NAMPT^high^* group compared to the *NAMPT^low^* group in *SPP1^+^* TAMs. Normalized enrichment scores (NES) are shown (left; adjusted *p* value<0.05). GSEA plots of selected top‐ranked Hallmark gene sets (right). NES and adjusted p values (Padj) are shown.

TCGA analysis indicated that *NAMPT* expression is upregulated in various cancer types compared to normal tissues (**Figure** **1**A). Several previous reports indicate that there is a statistically significant correlation between lower *NAMPT* mRNA expression and overall survival in colon and other cancer patients, thus suggesting that NAMPT may be an oncogenic factor that functions in the tumor progression process.^[^ ^13^, ^14^ ^]^ NAMPT in the tumor microenvironment (TME) might also contribute to cancer progression and metastasis since other cells within the microenvironment can interact with the tumor cells themselves to influence changes.^[^ ^4^ ^]^ We assessed the expression patterns of *NAMPT* in immune cell populations within tumor and adjacent normal tissues using publicly accessible 10X‐derived single‐cell RNA sequencing data obtained from colorectal cancer (CRC) patients. NAMPT mRNA expression in immune cell subpopulations of tumor tissues were comparable to those in adjacent normal tissues (Figure 1B). Notably, NAMPT was expressed predominantly in myeloid cell populations; among these, mast cell and dendritic cell types showed relatively lower expression of NAMPT than the remaining cell clusters (Figure S1A, Supporting Information). While excluding the two resident tissue macrophages, which showed preferential enrichment in normal mucosa versus tumors, we used three blood‐enriched clusters (i.e., CD14^+^/CD16^+^ monocytes), a tumor‐enriched *FCN1^+^* monocyte‐like cell cluster, and two tumor‐enriched tumor‐associated macrophage (TAM) clusters (i.e., *C1CQ^+^* and *SPP1^+^* TAMs) for further analyses (Figure 1C, left panel). *NAMPT* expression in macrophage clusters was comparable to that of monocyte clusters in adjacent normal tissues, however tumor tissues showed significant *NAMPT* upregulation in TAM clusters compared to monocyte clusters (Figure 1C, right panel). When *NAMPT* expression was further analyzed in two groups of TAMs in the tumor tissues, *SPP1^+^* TAMs showed a greater increase in *NAMPT* expression with respect to monocytes compared to *C1QC^+^* TAMs (Figure 1D). This is interesting because *SPP1^+^* macrophages are a unique subset of TAM associated with immunosuppressive activity, and have pro‐tumorigenic/pro‐metastatic roles in CRC.^[^ ^11^, ^12^ ^]^ We next divided *SPP1^+^* TAMs into *NAMPT^low^* and *NAMPT^high^* groups based on the median expression of *NAMPT* (Figure 1E). Differential expression analysis of the two groups revealed known mediators of M1/M2‐polarization of macrophages (M1/M2 TAMs) that were highly expressed in *NAMPT^high^* group (Figure 1F; Figure S1B, Supporting Information). Similar expression patterns of these selected genes were also observed in *C1CQ^+^* TAMs (Figure S1C,D, Supporting Information). Cell‐level module activity of these selected markers in M1/M2 TAMs was positively correlated with *NAMPT* expression and was highly enriched in *NAMPT^high^* group compared to *NAMPT^low^* group (Figure 1G). Gene ontology (GO) analyses of *NAMPT^high^ SPP1^+^* TAMs further showed enrichment of gene sets involved in epithelial to mesenchymal transition (EMT), the STAT3/5 signaling pathway, angiogenesis, and hypoxia response, among others. The activation of these processes might be involved in the heterogeneous phenotype and the functions of TAMs in response to various microenvironmental signals generated from tumor and stromal cells in the TME (Figure 1H). In contrast to GOs enriched in *NAMPT^high^ SPP1^+^* TAMs, the genes associated with the interferon (IFN)‐α response, PI3K/AKT/mTOR signaling pathway, reactive oxygen species generation, fatty acid metabolism, MYC targets V1, and DNA repair were highly enriched in *NAMPT^low^* group (Figure S1E, Supporting Information).

### 2.2 HIF‐1α Stabilization by NAMPT Promotes Angiogenic and Pro‐Tumoral Properties in Macrophages

**Figure 2:**
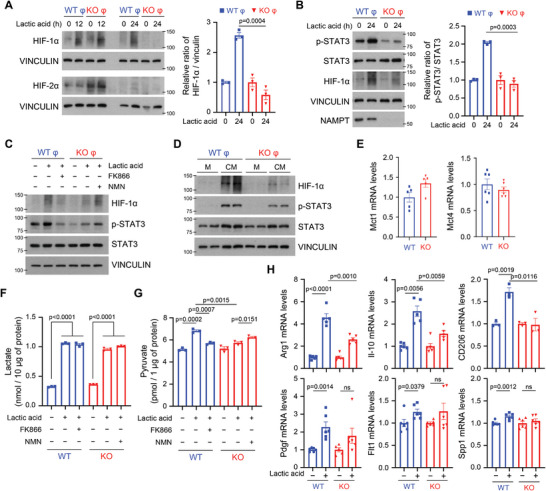
NAMPT potentiates the HIF‐1a/STAT signaling pathway when under lactic acidosis conditions. A,B) The expression levels of the indicated proteins were assessed in WT and *Nampt* KO macrophages treated with 15 mM lactic acid via western blotting (left). Quantification of the protein amounts is shown as a ratio of HIF‐1α to vinculin and p‐STAT3 to STAT3 (right). C) WT and *Nampt* KO macrophages were pretreated with 20 nM FK866 or 1 mM NMN for 6 h, followed by incubation with 15 mM lactic acid for 24 h. Cell lysates were subjected to western blotting. D) WT and *Nampt* KO macrophages were incubated with conditioned medium (CM) collected from MC38 cells for 24 h. The indicated proteins were analyzed by western blotting. E) mRNA levels of *Mct1* and *Mct4* genes in macrophages from WT and *Nampt* mKO mice were analyzed by quantitative real‐time PCR (qRT‐PCR). mRNA levels of *Mct1* and *Mct4* were normalized by mRNA level of *Tbp1*. F‐G) Concentrations of intracellular lactate (F) and pyruvate (G) were measured in WT and *Nampt* KO macrophages pretreated with 20 nM FK866 or 1 mM NMN for 6 h, followed by incubation with 15 mM lactic acid for 12 h. H) mRNA levels of angiogenesis‐ and M2 polarization‐related genes were quantified in WT and *Nampt* KO macrophages treated with 15 mM lactic acid using qRT‐PCR. mRNA levels of the indicated genes were normalized to the mRNA level of *Tbp1*. Results are represented as the mean ± SEM. Statistical analysis was performed using the unpaired two‐tailed Student's *t*‐test.

From the gene signature analysis, we hypothesized that TME could control the development of certain TAM populations and that NAMPT presence in macrophage might influence the TAM phenotype, which then could effectively remodel the CRC microenvironment. Interestingly, the HIF‐1α (hypoxia‐inducible factor‐1α) signaling pathway was significantly enriched in *NAMPT^high^SPP1^+^* TAMs compared with the *NAMPT^low^* group. This is significant since hypoxia is one of the important driving characteristics of alterations in TME.^[^ ^15^ ^]^ Tumor‐derived lactate has been reported to be sufficient to drive M2‐like TAM polarization in HIF‐1α‐dependent manner in TME.^[^ ^16^ ^]^ Consistent with this, addition of lactic acid induced a prolonged HIF‐1α (but not HIF‐2α) expression in *Nampt* wild type (WT), but this did not occur in *Nampt* deletion (KO) macrophages (**Figure** **2**A). In addition, lactic acid treatment increased protein expression of HIF‐1α without affecting its mRNA expression, indicating that NAMPT promotes protein stabilization of HIF‐1α (Figure S2, Supporting Information). Since the lactate‐mediated HIF‐1α/STAT3 signaling pathway is known to promote M2 polarization of macrophages in TME,^[^ ^17^ ^]^ we next examined whether HIF‐1α protein expression alters STAT3 signaling in these cells, as it is also involved in M2‐like TAM polarization.^[^ ^18^ ^]^ Phosphorylation of STAT3 was increased in WT macrophages in response to lactic acid (Figure 2B), correlating with the upregulated HIF‐1α expression. To explore whether the defects in HIF‐1α/STAT3 signaling result from deficiency of NAMPT, WT, or KO macrophages were treated with an inhibitor of NAMPT enzymatic activity, FK866, or the NAMPT product, NMN following the lactic acid treatment. Upregulated HIF‐1α and phosphorylated STAT3 were prevented by FK866 in WT macrophages, while NMN addition rescued these effects in the KO macrophages, suggesting that NAMPT expression controls the lactate‐mediated HIF‐1α/STAT3 signaling pathway in macrophages (Figure 2C). To support the idea that tumor cell‐derived lactate is sufficient to alter the HIF‐1α/STAT3 signaling pathway, conditioned medium (CM) from MC38 cells was applied to macrophages and upregulated HIF‐1α/phosphorylated STAT3 was observed in WT macrophages, indicating that NAMPT promotes this pathway (Figure 2D).

We investigated next whether defects in the HIF‐1α/STAT3 signaling pathway were caused by abnormalities in the lactate transport system in KO macrophages. The mRNA levels of lactate transporter genes, *Mct1* and *Mct4*, were similar between WT and KO macrophages, ruling out gene expression differences of these transporters (Figure 2E). The increased amounts of intracellular lactate upon exogenous lactic acid treatment in WT macrophages were also comparable to those in KO macrophages (Figure 2F). FK866 treatment did not reduce the intracellular lactate levels in WT macrophages and NMN did not alter the amounts in KO macrophages (Figure 2F). These data indicate that NAMPT expression and/or NAMPT enzymatic activity does not alter lactate transport. Intracellular lactate is converted to pyruvate^[^ ^19^ ^]^ which has been reported to alter HIF‐1α expression by inhibiting proline hydroxylase (PHD) activity.^[^ ^20^ ^]^ This process requires NAD, which is mainly supplied by NAMPT.^[^ ^21^ ^]^ We therefore hypothesized that *Nampt* deletion results in a reduction of lactate oxidation due to the insufficient supply of NAD. Lactic acid treatment did indeed increase pyruvate levels in WT macrophages, but not in KO macrophages (Figure 2G). Inhibition of NAMPT enzymatic activity reduced pyruvate levels in WT macrophages, while the addition NMN rescued pyruvate levels in KO macrophages (Figure 2G). These data indicate that NAMPT likely promotes HIF‐1α stabilization by increasing lactate oxidation. As hypoxic TME characterized by acidosis is known to polarize macrophages into immune‐suppressive or angiogenic M2‐like phenotypes, we next examined whether NAMPT contributes to gene expression of angiogenic and pro‐tumoral molecules in response to lactic acid treatment. In the presence of NAMPT, lactic acid treatment highly upregulated M2‐like phenotypes markers (*Arg1*, *Il‐10*, and *CD206*), and angiogenesis‐related molecules (*Pdgf* and *Flt1*) (Figure 2H). Since, the EMT hallmark gene SPP1 is significantly correlated with M2 polarization^[^ ^22^ ^]^ and since *NAMPT^high^ SPP1^+^* TAMs have shown that the enrichment of genes involved in EMT pathway, we expected that lactic acid would alter SPP1 expression in a NAMPT‐dependent manner. *SPP1* was indeed upregulated in WT macrophages in response to lactic acid (Figure 2H) indicating that NAMPT‐mediated HIF‐1α stabilization likely modulates the gene signature involved in M2‐like TAM polarization.

### 2.3 NAMPT Expression Skews Macrophages Toward an M2‐like Phenotype During Interactions Between Colon Cancer Cells and Macrophages

**Figure 3:**
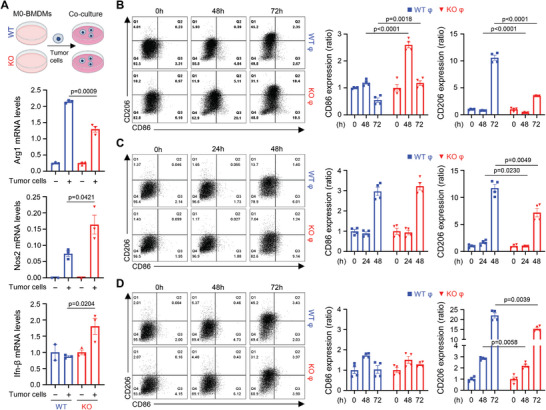
Macrophage NAMPT drives TAM polarity toward M2‐like phenotypes. A) mRNA of *Arg‐1*, *Nos2* and *Ifn‐β* were quantified in WT and *Nampt* KO macrophages co‐cultured with MC38 cells for 12 h. mRNA levels of the indicated genes were normalized to the mRNA level of *Tbp1*. B‐D) BMDMs were directly co‐cultured with tumor cells for the indicated times (B) and were co‐cultured with tumor cells using a transwell system for the indicated times (C). MC38 tumor‐conditioned medium was used as <3‐kDa fractions to stimulate macrophages for the indicated times (D). Representative flow cytometry plots (left) and bar graph (right). Results are represented as the mean ± SEM. Statistical analysis was performed using the unpaired two‐tailed Student's *t*‐test.

Crosstalk between macrophages and tumor cells via cell‐cell contact or via secreted factors remodels the CRC microenvironment.^[^ ^23^ ^]^ Our data derived under lactic acidosis conditions suggests that secreted factors (including lactic acid) from cancer cells may shape macrophage polarization status. To test whether NAMPT is involved in the polarization of TAMs caused by the interplay with tumor cells, we co‐cultured tumor cells with macrophages. When interacting with tumor cells the mRNA expression of the M2‐like TAM marker, *Arg1* was reduced, but the M1‐like TAM markers *Nos2* and *Ifn‐β* were potentiated in the KO cells compared to the WT cells (**Figure** **3**A), which is consistent with the lactic acid treatment data. FACS analysis showed that KO macrophages exhibited higher CD86 and lower CD206 expression than WT macrophages (Figure 3B). Bone marrow‐derived macrophages (M0) are known to differentiate into M1 or M2 macrophages in in vitro systems with LPS/IFN‐γ (M1) or interleukin‐4 (M2).^[^ ^24^ ^]^ Deficiency of NAMPT, however, did not alter M1/M2 polarization, indicating that NAMPT does not affect classical M1/M2 macrophage polarization (Figure S3A, Supporting Information). These data indicate that NAMPT deficiency might rather alleviate M2‐like TAM polarization via crosstalk with tumor cells. To further examine whether tumor‐secreted factors would affect the transition of TAMs phenotype according to the presence or absence of NAMPT expression, macrophages and tumor cells were cultured in a transwell system (Figure S3B,C, Supporting Information). Tumor‐secreted factors potentiated an M2‐like phenotype with a higher resultant CD206 in WT macrophages compared to KO macrophages, however, CD86 expression was similar in WT and KO macrophages (Figure 3C; Figure S3C, Supporting Information). Cell‐cell contact, or secreted factors from them, then, promoted polarization toward M2‐like phenotype in WT macrophages, however, the deficiency of NAMPT reduced this phenotype change. NAMPT previously promotes M2‐like TAM polarization in response to lactic acid treatment (see Figure 2). Lactic acid is known to be enriched in the fraction (<3 kDa) of tumor‐conditioned medium.^[^ ^25^ ^]^ Hence, we fractionated the tumor‐conditioned medium by size (<3 kDa) and found that the M2‐like TAM polarization was reduced in KO macrophages under incubation with the fraction compared to WT macrophages (<3 kDa) (Figure 3D; Figure S3D,E, Supporting Information).

NAMPT exhibits cytokine‐/adipokine‐like properties in the extracellular environment, where its enzymatic activity seems to be dispensable.^[^ ^26^ ^]^ It is now well established that NAMPT gene products are located both intracellularly (where it has an enzymatic function and is referred to as NAMPT) and extracellularly (where it has cytokine‐like functions and is referred to as visfatin/eNAMPT or PBEF) with widespread expression.^[^ ^8^ ^]^ Our data showed that deletion of *Nampt* within the myeloid compartment itself skews the macrophage population to M2‐like TAMs in the TME. However, this does not rule out the effect of extracellular NAMPT produced by other types of cells on tumorigenesis. To test the effect of visfatin/eNAMPT, we treated colon cancer cells (HT‐29 and HCT‐116) with visfatin and analyzed its effects on the colony‐forming ability and migration of these cells. As expected, visfatin treatment increased both of these activities, indicating that extracellular NAMPT could function as oncogenic factor in tumor cells (Figure S4, Supporting Information). As we previously showed that extracellular NAMPT does not affect macrophage polarization,^[^ ^27^ ^]^ we conclude that it must be intracellular NAMPT that is involved in TAM polarization in response to secreted factors, such as lactate, rather than extracellular NAMPT from tumor cells, which is removed from the conditioned medium by size exclusion (<3 kDa) (see Figure 3D). These results suggest that intracellular NAMPT in macrophages contributes to cell‐cell contact‐, or secreted factor‐mediated M2‐like TAM polarization in TME.

### 2.4 CRC Progression is Reduced in Mouse Models Upon Macrophage‐Specific Deletion of *Nampt*

**Figure 4:**
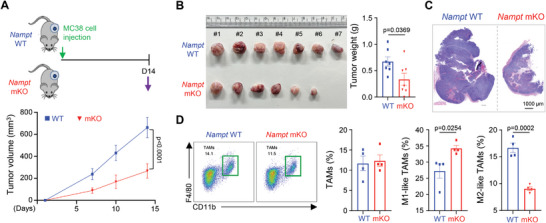
Macrophage‐specific deletion of *Nampt* alters TAM polarization in TME. A) MC38 cells were subcutaneously injected into WT (n = 7) and *Nampt* mKO (n = 7) mice (upper panel). Tumor diameters were measured at 7, 10 and 14 days after inoculation of MC38 cells (lower panel). B) Representative tumor images (left) and tumor weight (right) at day 14 in WT and *Nampt* mKO groups. C) Representative images of tumor tissues by H&E staining from WT and *Nampt* mKO mice. Scale bar = 1000 µm. D) The proportion of TAMs among CD45^+^ immune cells from WT and *Nampt* mKO groups (n = 4 per group) is shown (left). The proportion of CD86^high^ TAMs (M1‐like TAMs) and CD206^high^ TAMs (M2‐like TAMs) in WT and *Nampt* mKO groups (n = 4 per group) is shown (right). Results are represented as the mean ± SEM. Statistical analysis was performed using the unpaired two‐tailed Student's *t*‐test.

We have shown that NAMPT‐deficient macrophages lose their polarization toward the M2‐like phenotype in vitro; we next examined whether NAMPT deficiency affects tumor progression through macrophage polarization in vivo. Transplantable MC38 mouse colon cancer cells and mice carrying a specific deletion of the *Nampt* gene in the myeloid compartment (*Nampt^f/f^ LysMCre^+/−^*; mKO) were used to test myeloid‐specific activity of NAMPT in tumor progression. There was no difference in levels of serum alanine aminotransferase (ALT) and aspartate aminotransferase (AST) between WT and mKO mice inoculated MC38 cells (Figure S5A, Supporting Information). However, when compared with the WT, mKO mice showed a reduced tumor volume/weight suggesting that NAMPT expression/enzymatic activity may promote tumor progression in TME (**Figure** **4**A‐C). To support this hypothesis, we analyzed the tumor‐associated macrophages phenotypes from isolated macrophages in the tumor samples (Figure S5B, Supporting Information). The number of F4/80^+^ macrophages were similar in tumor tissues from WT and mKO mice, however, the population of CD86^high^M1‐like TAMs was higher in tumor tissues from mKO mice than in WT mice, while the population of CD206^high^M2‐like TAMs was much higher in tumor tissues from WT mice (Figure 4D). Consistent with the reduced population of CD206^high^ TAMs, the amount of *Arg1* mRNA was reduced in tumor tissues from mKO mice compared to WT mice, although we did not detect a difference in the mRNA expression of *Nos2* in tumor tissues from these mice (Figure S5C, Supporting Information). This indicates that NAMPT inhibition reduces tumor outgrowth by limiting the population of immunosuppressive tumor‐promoting M2‐like TAMs in TME.

**Figure 5:**
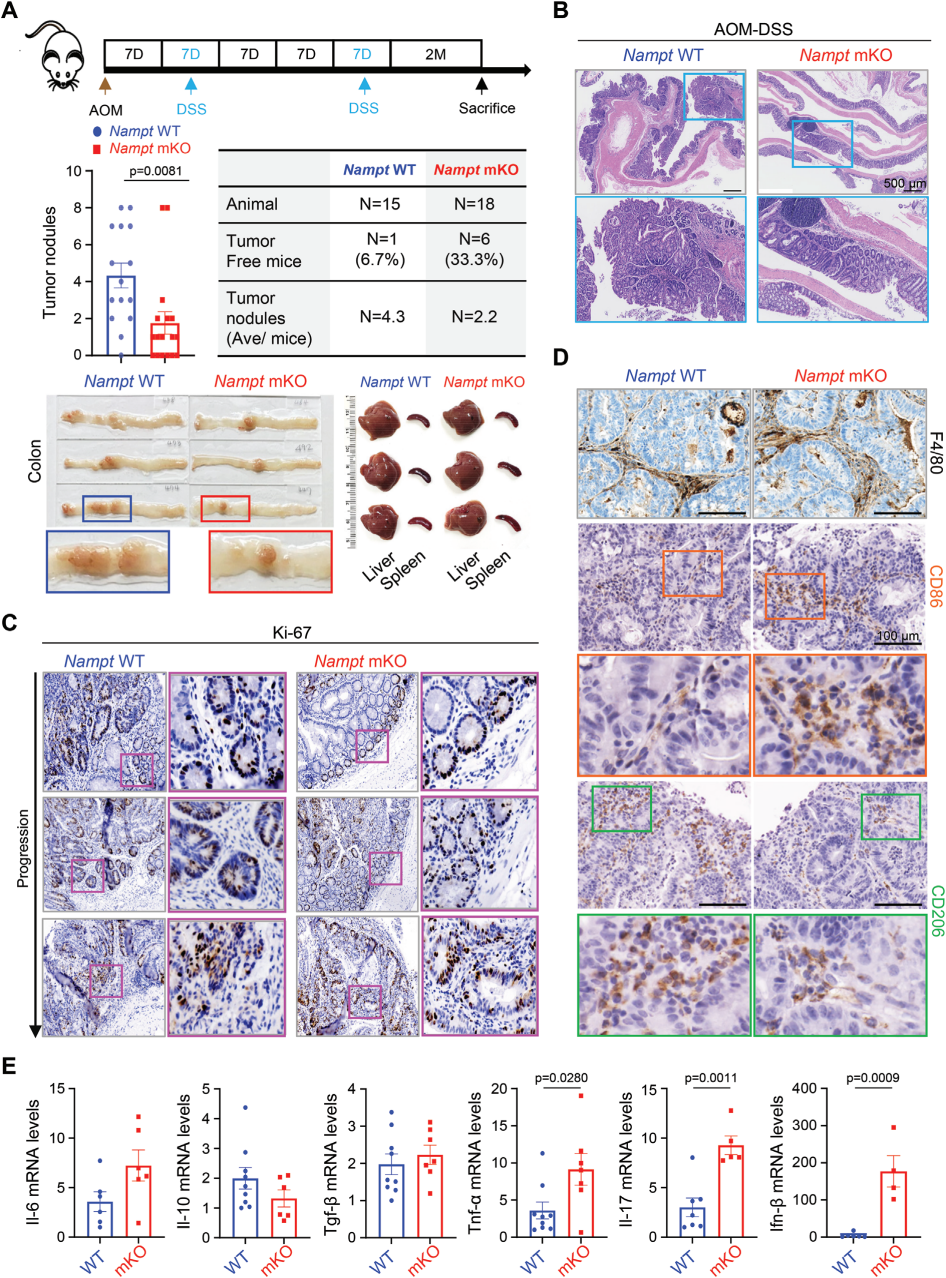
Macrophage‐specific deletion of *Nampt* attenuates colitis‐induced tumorigenesis. A) Groups of WT and *Nampt* mKO mice were subjected to the AOM/DSS‐induced CRC model. A schematic representation of the AOM/DSS treatment is shown (upper panel). Number of tumor nodules and percentage of tumor‐free mice in AOM/DSS‐treated WT (n = 15) and *Nampt* mKO (n = 18) mice are shown (middle panel). Representative images of colonic tissues, liver, and spleen tissues from WT and *Nampt* mKO mice treated with AOM/DSS (lower panel). B) Representative images of H&E staining of colonic tumor tissues are shown. Scale bar = 500 µm. C,D) Immunohistochemistry of Ki‐67 (C), F4/80, CD86 and CD206 (D) in colonic tumor tissues from WT and *Nampt* mKO mice treated with AOM/DSS. Scale bar = 100 µm. E) Relative mRNA levels of cytokines in colonic tumor tissues from WT and *Nampt* mKO mice treated with AOM/DSS are shown. Results are represented as the mean ± SEM. Statistical analysis was performed using the unpaired two‐tailed Student's *t*‐test.

Considering the anti‐tumoral immunity subsequent to the inhibition of myeloid NAMPT, we next investigated its influence on CRC progression using an AOM/DSS‐induced colon cancer model. The mKO mice showed reduced numbers of tumor nodules and incidence of tumorigenesis when compared with WT mice, with no signs of differences in liver, spleen and serum (**Figure** **5**A; Figure S6A‐C, Supporting Information). H&E staining indicated that there were no differences in histological structures in colon tumor regions in the WT and mKO mice (Figure 5B; Figure S6D, Supporting Information). Ki‐67 expression in colonic tumor tissues from WT or mKO mice showed little difference in tumor cell regions, but overall, colonic tumor tissues from mKO showed less Ki‐67 positive cells in certain areas (Figure 5C; Figure S6E, Supporting Information). When examining the distribution of TAMs within colon tumor tissues, we found that F4/80^+^ macrophages infiltrated within the colonic tumor tissues similarly between WT and mKO mice (Figure 5D, upper panel). However, higher populations of CD86‐expressing cells and lower populations of CD206‐expressing cells were observed in colonic tumor tissues from mKO mice (Figure 5D, middle and bottom panel; Figure S6F, Supporting Information). Furthermore, the mRNA expression of *Tnf‐α*, *Il‐17* and *Ifn‐β* were higher in colonic tissues from mKO mice (Figure 5E); FACS analysis indicated a reduced M2‐like TAM population in mKO mice compared to WT mice (Figure S6G, Supporting Information). Taken together, our data suggest that the NAMPT expression in macrophages enhances the development of pro‐tumoral TAMs in TME resulting in potentiation of CRC progression.

### 2.5 NAMPT Provides a Favorable Microenvironment for the Pro‐Tumoral Macrophages through Efferocytosis Activity in TME

**Figure 6:**
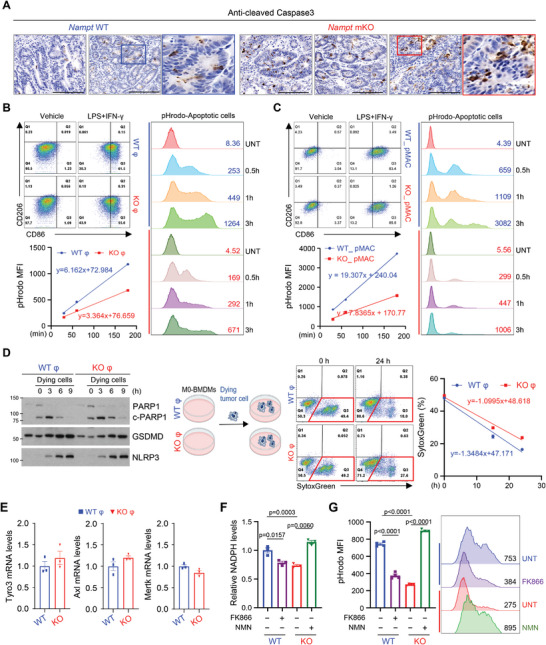
NAMPT in macrophages is required for efficient clearance of apoptotic tumor cells. A) Paraffin‐embedded colonic tumor sections from AOM/DSS‐treated mice were stained with anti‐cleaved caspase3. Scale bar = 100 µm. B,C) Flow cytometry analysis of CD86 and CD206 expressions in CD11b^+^F4/80^+^ macrophages upon LPS/IFN‐γ treatment (left upper). The phagocytic activity of BMDMs (B, left bottom) and pMAC (C, left bottom), treated with LPS/IFN‐γ for 12 h was measured from 30 min to 180 min after adding dying MC38 cells labeled with pHrodo green dye using flow cytometry. Representative histogram of pHrodo intensity is shown (B and C, right).D) Western blot analysis of co‐culture experiment of BMDMs with dying MC38 cells for different time points (left). Flow cytometry analysis of SytoxGreen‐stained population during co‐culture of BMDMs and dying MC38 cells (right). E) Relative mRNA levels of *Tyro3, Axl, Mertk* genes in BMDMs from WT and *Nampt* mKO mice. F) NADPH levels are measured in WT and *Nampt* KO BMDMs treated with FK866 or NMN. G) BMDMs were treated with LPS/IFN‐γ for 12 h in the presence or absence of FK866 or NMN. The phagocytic activity of BMDMs was measured 2 h after adding dying MC38 cells labeled with pHrodo green dye by using flow cytometry (left). Representative histogram of pHrodo intensity is shown (right). Results are represented as the mean ± SEM. Statistical analysis was performed using the unpaired two‐tailed Student's *t*‐test.

Cell death is a common event in solid tumors during malignant process; corpse clearance, which is referred to as “efferocytosis”, has important effects on immunosuppression.^[^ ^28^ ^]^ Defects in efferocytosis contributes to anti‐tumor immunity via increasing population of M1‐like TAMs and cytotoxic T cells in TME.^[^ ^29^, ^30^ ^]^ Since our data show a relatively high population of M1‐like TAMs in tumor tissues from mKO mice this may indicate that NAMPT deficiency leads to defects in efferocytosis compared to cells with high expression of NAMPT. We examined whether the tumor tissues from mKO mice have more apoptotic cells than WT. Apoptotic cells were more predominant in tumor tissues from mKO mice compared to WT mice as measured by IHC staining with cleaved caspase‐3 (**Figure** **6**A). To test that whether this increased population of apoptotic cells in TME is due to the defect of efferocytosis, we examined the efferocytosis activity in macrophages. To do this, we first prepared apoptotic cells via treatment of etoposide in MC38 and CT26 cells (Figure S7A,B, Supporting Information). When macrophages from WT and *Nampt* mKO mice were challenged with apoptotic cells that had been conjugated to pHrodo green dye, KO macrophages showed a reduced green intensity indicating reduced efferocytosis activity (Figure 6B; Figure S7B, Supporting Information). To confirm this phenomenon in a different type of macrophages, we first checked whether peritoneal macrophages (pMACs) respond similarly to in‐vitro M1/M2 polarization by LPS/IFN‐γ or IL‐4 treatment. No observable differences in M1/M2 polarization were shown in the presence/absence of Nampt expression in pMACs, similar to the results in the bone marrow derived macrophages (BMDMs) (Figure S7C,D, Supporting Information). Consistent with BMDMs, pMAC from *Nampt* mKO mice showed a reduced phagocytotic activity suggesting that NAMPT is required for efferocytosis activity of macrophages in general (Figure 6C). A defect of efferocytosis activity in KO macrophages was further confirmed by delayed degradation of cleaved PARP and more intense SytoxGreen‐positive signals derived from dead MC38 cells (Figure 6D). To rule out that reduced efferocytosis activity in KO macrophages was due to a defect in the recognition of phosphatidylserine on apoptotic cancer cells by TAMs, we measured mRNA levels of receptor tyrosine kinase family members such as *MerTK*, *Tyro3*, and *Axl*. Deficiency of NAMPT did not alter TAM receptor tyrosine kinase expression (Figure 6E). Furthermore, NMN treatment rescued efferocytosis activity in KO macrophages via increasing NADPH levels (Figure 6F,G), indicating that NAMPT likely promoted efferocytosis activity via an increase in NADPH during tumor progression.

Next, we investigated whether defects of efferocytosis were correlated with macrophage polarization status. When apoptotic cells were added to macrophages, KO macrophages showed increased mRNA of M1‐related genes, but reduced mRNA of M2‐related genes compared to WT macrophages (Figure S7E,F, Supporting Information). Collectively, the results support the notion that macrophage‐specific ablation of *Nampt* attenuates the clearance of apoptotic cancer cells and that defects of efferocytosis activity might provide an adverse environment for M2‐like TAMs.

### 2.6 Type I IFN Responses Promoted via the STING Pathway Contribute to Anti‐Tumoral Immunity of NAMPT‐Deficient Macrophages

A recent study showed that blocking phagocytic clearance of apoptotic cells increased the release of cyclic GMP‐AMP (cGAMP) from dying tumor cells and induced a stimulator of interferon genes (STING)‐dependent type I IFN response.^[^ ^29^ ^]^ We investigated whether defects in efferocytosis activity in NAMPT‐deficient TAMs could regulate cyclic GMP‐AMP synthase (cGAS)‐STING signaling. Activated STING signaling and type I IFN‐response genes were confirmed in M0 macrophages upon cGAMP treatment (Figure S8A, Supporting Information), however there was no difference between WT and *Nampt* KO BMDMs in the activation of STING upon cGAMP treatment (Figure S8B,C, Supporting Information).

**Figure 7:**
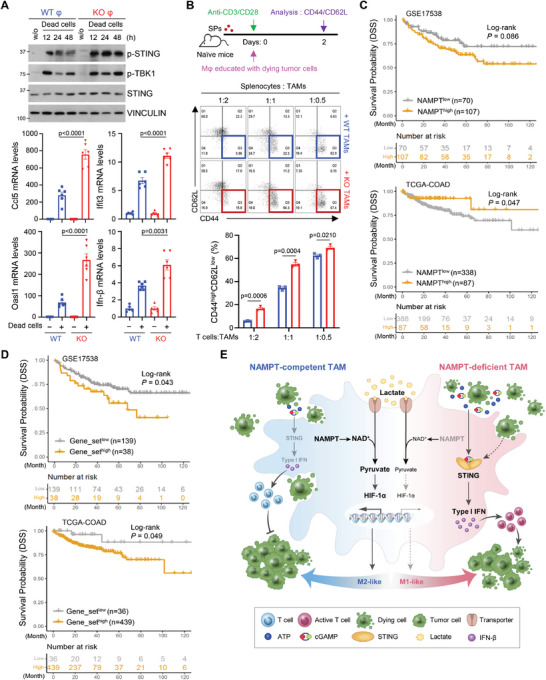
Enhanced STING‐dependent type I IFN responses promote cytotoxic T cell activity in NAMPT‐deficient macrophages. A) BMDMs from WT and *Nampt* mKO mice were treated with dying MC38 cells for the indicated times. The indicated proteins were analyzed by western blotting (upper) and mRNA levels of *Ifn‐β* and IFN response genes were analyzed by qRT‐PCR (bottom). mRNA levels of the indicated genes were normalized to the mRNA levels of *Tbp1*. B) A Schematic diagram of co‐culture with TAMs and splenocytes. To obtain the trained TAMs, BMDMs were cultured with dying tumor cells for 48 h. TAMs were incubated with splenocytes from naïve mice for 2 days. FACS analysis of the proportion of effector cells (CD44^high^CD62L^low^) of CD8^+^ T cells co‐cultured with TAMs is shown. Results are represented as the mean ± SEM. Statistical analysis was performed using the unpaired two‐tailed Student's *t*‐test. C) Disease‐specific survival Kaplan‐Meier (KM) curves showing *NAMPT^low^* (grey) and *NAMPT^high^* (yellow) groups in a colon cancer patient cohort (top: GSE17538, bottom: TCGA‐COAD). The log‐rank p‐value (P) and the number of patients successfully stratified (n) as determined from univariate Cox regression analysis are shown. D) Disease‐specific survival Kaplan‐Meier (KM) curves showing the two groups stratified by gene signatures (n = 55) highly enriched in *NAMPT^high^* TAMs (GSE146771) in a colon cancer patient cohort (top: GSE17538, bottom: TCGA‐COAD. The log‐rank p‐value (P) and the number of patients successfully stratified (n) determined from univariate Cox regression analysis are shown. E) Diagram for reprogramming of NAMPT‐dependent TAM phenotype in TME.

We next examined whether compromised removal of dying cells could potentiate STING signaling. To do this, M0 macrophages were cultured with dying cancer cells. We found that dying cancer cells could activate STING signaling and type I IFN‐response genes in macrophages (Figure S8D, Supporting Information). NAMPT‐deficient macrophages with resultant compromised efferocytosis activity had more potent activation of STING signaling, and type I IFN‐response genes compared to WT macrophages (**Figure** **7**A; Figure S8E, Supporting Information). STING‐signaling in immune cells promotes type I IFN‐dependent spontaneous T‐cell priming that increases tumor immunogenicity and improves cancer immunotherapy.^[^ ^31^ ^]^ To investigate whether NAMPT‐deficient TAMs improved T‐cell function, activated splenocytes were cultured with TAMs (generated via co‐culture with dying cancer cells). *Nampt* KO TAMs increased the population of effector CD8 T cells (CD44^high^CD62L^low^) compared to WT TAMs (Figure 7B). However, no difference in the proportion of effector T cells was observed upon co‐culture of M0 macrophages from both WT and *Nampt* KO, indicating that impairment of efferocytosis by NAMPT deficiency enhanced anti‐tumor T cell responses (Figure S8F, Supporting Information). Taken together, our data suggest that NAMPT deficiency in macrophages drive M1‐like TAM polarization and provide anti‐tumor activity via potentiated cytotoxic T‐cell activity in TME.

## 3 Discussion

While several previous studies of NAMPT in cancer have been mainly focused on its upregulated expression level in tumor cells, it has been less clear as to whether NAMPT expression in TME affects CRC progression. We demonstrate here that NAMPT deficiency in macrophages attenuates tumor progression in colon cancer models. *Gerner* et al. revealed that FK866 treatment ameliorated AOM/DSS‐induced colon cancer and proposed the possibility that FK866's anti‐inflammatory potential was due to effects on macrophages.^[^ ^32^ ^]^ However, FK866 treatment targets other cells in the TME and mediates extraintestinal effects. Our study provides evidence of a crucial function of NAMPT in reprogramming TAMs in response to tumor microenvironmental factors.

NAMPT was originally discovered as a pre‐B‐cell colony‐enhancing factor (PBEF), while other groups also discovered it as an adipokine, visfatin.^[^ ^33^ ^]^ Recent reports suggested that extracellular NAMPT from the macrophages stimulates myoblast proliferation to promote muscle regeneration in mice via the NAMPT‐CCR5 axis;^[^ ^34^ ^]^ a more directed mode of action of NAMPT has also previously been shown to induce endothelial cell proliferation.^[^ ^35^ ^]^ *Audrito* et al. reported that chronic lymphocytic leukemia (CLL) lymphocytes produced extracellular NAMPT, which promotes differentiation of CLL monocytes into M2 macrophages independent of the enzymatic function of the molecule.^[^ ^26^ ^]^ However, it is still unclear as to the receptor and the downstream signaling events for this extracellular form.^[^ ^21^ ^]^ Rediscovery of the intracellular form of NAMPT as the key enzyme in NAD formation has considerably widened its potential roles in immunity, metabolism, and cancer.^[^ ^21^, ^36^ ^]^ In the field of cancer biology, NAMPT has been considered as a potent oncogenic factor in colon cancer, breast cancer, and non‐small‐cell lung cancer.^[^ ^13^, ^14^, ^37^ ^]^ In CRC progression, NAMPT increases tumorigenicity by inducing cancer stem cell‐like properties through PARP/sirtuin1 (Sirt1)^[^ ^14^ ^]^ and inhibition of NAMPT suppresses cell growth via the Sirt1/p53 signaling pathway.^[^ ^38^ ^]^ NAMPT also promotes tumor growth and invasiveness by regulating autophagy via mTOR pathway in breast cancer cells and functions as prognostic indicator in patients with triple‐negative breast cancers.^[^ ^13^ ^]^ In contrast, a recent study showed that NAMPT deficiency restrained MDSC (myeloid‐derived suppressor cells) mobilization from bone marrow to the periphery, leading to the enhanced antitumor activity.^[^ ^39^ ^]^ Its broad spectrum of expression in various cells is mirrored by its diverse effects on tumor progression in the TME. In this study, we identified a specific function of macrophage NAMPT during CRC progression, although not excluding other roles for NAMPT expression in the other components of the TME.

Lactate accumulation is a hallmark of solid cancers but has been considered a metabolic waste product of glycolytic tumors.^[^ ^40^ ^]^ However, it is becoming increasingly recognized as an important TME signal that is responsible for regulating the effector functions of a variety of tumor‐infiltrating immune cells.^[^ ^41^ ^]^ Tumor‐derived lactate was shown to be sufficient to drive macrophage M2 polarization in a HIF‐1α‐dependent manner,^[^ ^16^ ^]^ although the metabolic mechanism involved has not yet been elucidated. We demonstrate here that the molecular mechanism for engagement of NAMPT in promoting M2‐like TAM polarization in response to tumor‐derived lactate is via HIF‐1α stabilization through providing a NAMPT‐dependent sufficient NAD supply for lactate to pyruvate conversion. In addition, increased HIF‐1α is correlated with phosphorylation of STAT3, an oncogenic signaling pathway that has important roles in M2‐like TAM polarization,^[^ ^18^ ^]^ suggesting that macrophage NAMPT function as an upstream regulator in this process.

Our data point to the activation of the STING pathway as being important for controlling anti‐tumor immunity via increasing IFN response genes in NAMPT‐deficient TAMs in response to the engulfment of dying tumor cells. Active clearance of dying cells by phagocytes, referred to as efferocytosis generates a tumor‐tolerant, immunosuppressive TME.^[^ ^28^ ^]^ Efferocytosis inhibition via treatment with blocking antibodies for phagocytic receptors, MerTK and Axl, or impairment of LC3‐associated phagocytosis in TAMs have been reported to engage STING‐dependent type I IFN production.^[^ ^29^, ^30^, ^42^ ^]^ Consistently, we found that STING activation was potentiated in KO macrophages, which have defects in clearing apoptotic cells, although we did not rule out whether there is difference in tumor‐derived cGAMP or defects in phagocytosis‐dependent lysosomal fusion in our experimental system. Our data show that NAMPT‐deficient TAMs initiate a higher levels of effector T cells (CD44^high^CD72L^low^), suggesting that inhibition of NAMPT improves anti‐tumor immunity by enhancing cytotoxic T cell function. A significant finding in the analysis of scRNA‐seq data is that *SPP1+*TAMs, which are known to contribute to poor prognosis in CRC patients, exhibit high levels of *NAMPT* expression. Survival analyses of bulk transcriptomic data from microarray (GSE17538) and RNA‐seq (TCGA‐COAD) datasets reveal heterogeneous results for the expression level of *NAMPT* alone. GSE17538 shows non‐significant results, while TCGA‐COAD demonstrates a prognostic significance with a minimal statistical level (Figure 7C). These conflicting findings across different datasets may be attributed to the amalgamation of NAMPT expression signals originating from various cell types within the tumor microenvironment, rather than NAMPT within the TAMs themselves. On the other hand, the *NAMPT^high^* TAMs‐enriched gene set was correlated with a worse prognostic outcome in CRC patients using the microarray dataset (Figure 7D, top), which was further supported by the analysis of TCGA‐COAD dataset (Figure 7D, bottom). Thus, we propose that the collective expression level of NAMPT‐derived TAM‐specific genes may accurately predict poor CRC patient outcomes, and considering TME components may provide more precise information for predicting of patient survival or their response to therapy. Overall, our study suggests a pivotal role of NAMPT in TAM reprogramming by regulating the HIF‐1α/STAT3 and a reduction in STING activation (Figure 7E). These molecular mechanisms imply that inhibition of NAMPT enzymatic activity or expression in TME components could possibly be a viable therapeutic strategy for CRC patients.

## 4 Experimental Section

### Single‐Cell RNA Sequencing Analysis

The 10X‐derived TPM‐normalized expression values and the associated cell‐level metadata including the authors’ defined cell type annotation were obtained from the NCBI GEO under the accession code GSE146771.^[^ ^11^ ^]^ A standard workflow provided by the Seurat package (version 4.1.1) was applied in R (version 4.2.1) to perform feature selection, data scaling, dimensional reduction by principal component analysis (PCA), and cell clustering. Uniform Manifold Approximation and Projection (UMAP) reduction was used to visualize and analyze cells. Cells of blood samples were excluded from further analyses. To compute the cell‐level module activity of *CCL20, CXCL8, IL1B, CXCL3, CXCL2*, and *VEGFA*, the AddModuleScore function provided by the Seurat package was used. The gene signature (n = 55) enriched in *NAMPT^high^* TAMs as compared to *NAMPT^low^* TAMs was derived from FindMarker function provided by the Seurat package (logFC>0.6 and padj<0.05). Differential expression (DE) analysis, gene ontology (GO) analysis, and gene set enrichment analysis (GSEA) were performed using the msigdbr (version 7.5.1), fgsea (version 1.22.0), dplyr (version 1.0.10), presto (version 1.0.0), and ggplot2 (version 3.3.6) packages in R.

### TCGA Data Analysis

For pan‐cancer analysis of *NAMPT* expression across different cancer types, TIMER2.0^[^ ^43^ ^]^ was used to compare the expression levels of tissue‐level normal and tumor tissues derived from The Cancer Genome Atlas (TCGA). For survival analysis, patients were stratified into two groups using cutoff values obtained from Cutoff Finder^[^ ^44^ ^]^ with the most optimal log‐rank p values.

For *NAMPT* expression, TCGA‐COAD gene expression data [RNA (Final)‐EBPlusPlusAdjustPANCAN_IlluminaHiSeq_RNASeqV2.geneExp.tsv] were downloaded from NCI's Genomic Data Commons (GDC) (https://gdc.cancer.gov/about‐data/publications/pancanatlas) and Log2(normalized counts+1) was used as input into Cutoff Finder. For the gene signature (n = 55) enriched in *NAMPT^high^* TAMs as compared to *NAMPT^low^* TAMs in GSE146771, the aggregated enrichment score was computed via the ssGSEA function^[^ ^45^ ^]^ using the matrixStats package (version 1.0.0) in R. This enrichment score was then used as input for the Cutoff Finder tool. TCGA‐COAD survival data was downloaded from UCSC Xena Browser.^[^ ^46^ ^]^

### Microarray Data Analysis

The RMA‐normalized expression data from human tumor colorectal cancers (GSE17538‐GPL570) were loaded into R using the GEOquery package (version 2.64.2). The median of mean of log2‐transformed expression levels of *NAMPT* mapped array probes (“1 555 167_s_at”, “217 739_s_at”, “243 296_at”) was used as a cutoff to stratify the patient cohort into *NAMPT^low^* and *NAMPT^high^* groups. For the gene signature (n = 55) enriched in *NAMPT^high^* TAMs as compared to *NAMPT^low^* TAMs in GSE146771, the aggregated enrichment score was computed via the ssGSEA function^[^ ^45^ ^]^ using the matrixStats package (version 1.0.0) in R. This enrichment score was then used as input for the Cutoff Finder tool^[^ ^44^ ^]^ to obtain the cut‐off score, with the most optimal log‐rank p values. Patients were then stratified into two groups using this cutoff score. Kaplan–Meier survival curves were derived for overall survival and disease‐specific survival endpoints using the survival (version 3.3.1) and survminer (version 0.4.9.999) packages in R.

### AOM‐DSS‐Induced Colorectal Cancer

*Nampt flox/flox* and *LysM‐cre* mice on a C57BL/6 background have been previously described.^[^ ^27^ ^]^ Mice were maintained according to the guidelines of the Institutional Animal Care and Use Committee, who approved all animal procedures (2020–0013). As previously reported,^[^ ^47^ ^]^ age‐ and sex‐matched wild type and mKO mice were injected intraperitoneally with 10 mg k^−1^g AOM (Sigma). After 7 days, 2% DSS (MP Biomedicals) was given in the drinking water for 7 days, followed by regular drinking water for 2 weeks. This cycle was repeated 2 times, and the mice were sacrificed 90 days after AOM injection.

### Xenograft Tumor Model

Age‐ and sex‐matched *Nampt^f/f^* and *Nampt^f/f^LyzMCre^+/−^* mice were inoculated subcutaneously with 5 × 10 ^5^ MC38 cells into the right rear flank. Tumor sizes were measured 1 week after tumor inoculation using digital vernier calipers. Mice were sacrificed 14 days after tumor cell injection and tumors were excised and processed for other experiments.

### Tumor Digestion

Tumors were dissected and dissociated in RPMI medium with 3% FBS, 10 mM HEPES (Gibco), 100 µg/mL penicillin–streptomycin (Gibco), 25 µg/mL liberase TL (Roche), 200 U/mL DNase I (Roche) and 1 mg/mL collagenase D (Roche) for 30 min at 37°C with agitation, followed by treatment with ACK lysing buffer (Gibco) for red blood cell (RBC) lysis, and strained through a 70 µm strainer to remove undigested tumor tissues.

### Cell Culture and Isolation of Bone Marrow‐Derived Macrophages and Peritoneal Macrophages

Bone marrow cells were flushed from dissected femurs of 8–10‐week‐old C57BL/6 mice and differentiated in RPMI medium containing 10% heat‐inactivated fetal bovine serum, 10 mm HEPES, 2 mM L‐glutamine, 10 mM MEM non‐essential amino acids solution, and penicillin/streptomycin with 20 ng mL^−1^ M‐CSF (Peprotech) for 5–7 days. Peritoneal macrophages (pMAC) were harvested from mice treated with an intraperitoneal injection of 3% Brewer thioglycollate medium and adherent pMAC were cultured with 20 ng mL^−1^ M‐CSF. MC38 cells were purchased from Kerafast and CT26 cells were obtained from ATCC. They were cultured in RPMI plus 10% FBS, and penicillin/streptomycin at 37 °C with 5% CO_2_.

### Co‐Culture Experiments

For in vitro co‐culture of BMDMs and tumor cells, 4 × 10^5^ BMDMs were plated per well in six‐well plate in 1.5 mL of macrophage medium. On the next day, cancer cells were harvested with trypsin‐EDTA and subsequently resuspended them in macrophage medium. MC38 (1 × 10^5^) cells per six well plates were then added. In co‐culture with dying tumor cells, MC38 cells were treated with 150 µm etoposide (Sigma) for 18 h and cells were centrifuged at 2000 rpm for 10 min. Dying cells were washed twice with PBS and then were incubated with BMDMs. After the indicated times, the cells were harvested and analyzed. In co‐culture of BMDMs and tumor cells by using transwell system (3412, Corning incorporated), BMDMs (4 × 10^5^) per well were plated in six‐well plate in 1.5 mL of macrophage medium (bottom) and plated 2 × 10^5^ MC38 cells or CT26 cells per upper well. After the indicated times, the cells were harvested and analyzed.

For in vitro co‐culture of splenocytes and TAMs, spleens from WT mice were isolated and passed through 70 µm filters to generate a single‐cell suspension. After red blood cell lysis, splenocytes were plated in complete RPMI medium supplemented with 0.05 mm β‐mercaptoethanol in 12 well plates (5 × 10^5^ cells per well) coated with 1 µg mL^−1^ anti‐CD3ε (145‐2C11) (Biolegend) and 2 µg mL^−1^ anti‐CD28 (37.51) (Biolegend) antibodies. Co‐cultured TAMs with dying MC38 cells (prepared as described above) were added at the indicated ratios, and the plates were incubated at 37°C. After 48 h, the cells were harvested, and assessed the indicated surface marker levels by flow cytometry.

### Flow Cytometry Analysis

Cell suspensions were stained on ice for 20 min in the dark with various combinations of fluorochrome‐conjugated antibodies, including anti‐CD45 (30‐F11), anti‐CD11b (M1/70), anti‐F4/80 (BM8), anti‐CD206 (MMR), anti‐CD86 (GL‐1), CD4(GK1.5), CD8(53‐6.7), CD44(IM7) and CD62L(MEL‐14) (all from BioLegend). Samples were acquired on a FACS Canto II flow cytometer (BD). Data were analyzed using the FlowJo Software (BD).

### Histology and Immunohistochemistry

Tumors were fixed in 4% paraformaldehyde solution overnight at 4°C. Paraffin‐embedded sections were cut at a thickness of 5 µm and stained with H&E solution. Immunohistochemistry was performed using rabbit anti‐Ki‐67 (ab16667, Abcam), rabbit anti‐cleaved caspase3 (9661, Cell Signaling Technology), rat anti‐F4/80 (ab6640, Abcam), rabbit anti‐CD86 (19589, Cell Signaling Technology) and anti‐CD206 (24595, Cell Signaling Technology) antibodies.

### Western Blotting

Cells from each group were lysed using M2 buffer (20 mm Tris at pH 7, 0.5% NP‐40, 250 mm NaCl, 3 mm EDTA, 3 mm EGTA, 2 mm DTT, 0.5 mm PMSF, 20 mm β‐glycerol phosphate, 1 mm sodium vanadate, and 1 mg mL^−1^ leupeptin) and mice tissues were lysed using a lysis buffer composed of 50 mm Tris‐HCl (pH 7.5), 150 mm NaCl, 50 mm NaF, 1% Tween 20, 0.2% NP‐40 and protease inhibitors on ice for 20 min. Equal amounts of cell and tissue extracts were separated by SDS‐PAGE. Proteins were detected using the following antibodies. Primary antibodies: anti‐NAMPT (Bethyl Laboratories, A300‐372A), anti‐Vinculin (Sigma, V9131), and anti‐GSDMD (Abcam, ab225867). Anti‐CD86 (19589), anti‐p‐STING (72971), anti‐p‐TBK1 (5483), anti‐p‐IRF3 (4947), anti‐p‐p65(3033), anti‐p‐STAT3(Y705) (9145), anti‐STAT3 (4904), anti‐HIF‐1α (36169), anti‐HIF‐2α (57921), anti‐PARP‐1 (9542), and anti‐STING (13647) were from Cell Signaling Technology. Following the incubation with HRP‐conjugated secondary antibody (Jackson ImmunoResearch), detection was performed using enhanced chemiluminescent substrate (Thermo Scientific).

### Quantitative RT‐PCR

RNA was extracted using the TRIzol reagent (Life Technologies). cDNA was generated using MMLV reverse transcriptase (Promega) with 1 µg of total RNA and oligo(dT) primer. Equal amounts of cDNA product were used in real‐time PCR with GoTaq qPCR Master Mix (Promega). Gene expression was normalized to that of TBP1. Real‐time PCR was performed on CFX Connect. The oligonucleotides are listed in the Table S1 (Supporting Information).

### Efferocytosis Assay

MC38 cells or CT26 cells were treated with 150 µm etoposide for 18 h (to prepare dying cells) then cells were incubated with pHrodo iFL Green STP ester (Invitrogen) following the manufacturer's protocols. The labeled cells were added to BMDMs for 30 min. Fresh macrophage medium was added to BMDMs, and engulfment was monitored for the indicated time by FACS analysis (Canto II flow cytometer, BD).

### Measurement of Acetyl‐CoA, Lactate, and NADPH

Bone marrow‐derived macrophages (BMDMs) were harvested and analyzed using PicoProbeAcetyl‐CoA Fluorometric Assay Kit (Biovision), EnzyFluo L‐Lactate Assay Kit (Bioassay), and Elite NADPH Assay Kit (eEnzyme) according to the manufacturer's instructions.

### Statistical Analysis

Data were analyzed using the unpaired two‐tailed Student's *t*‐test with GraphPad Prism 9 and were presented as the mean ± standard error of the mean (SEM). Statistical significance was set at p < 0.05.

## Conflict of Interest

The authors declare no conflict of interest.

## Author Contributions

S.M.H. and A‐Y. L. contributed equally to this work. All authors are responsible for the overall content as the guarantors. S.M.H. and A‐Y.L. performed most of the experiments and analyzed the data. B.‐J.K., J.‐E.L., S.‐Y.S., Y.‐J.H., and J.‐H.C. provided technical support in the context of the in vivo model and intellectual support for data analysis. S.B.L. and J.T.N. analyzed publicly available data from CRC patients and G.Y. contributed to discussion of data. D.L. analyzed immunohistochemistry data and provided technical support in the context of immunohistochemistry data performed pathological analysis. Y.‐S.K. and M.J.M. wrote/edited the manuscript. Y‐S.K. supervised the study and took responsibility for all of the data. All authors have read and approved the final manuscript.

## Supporting information

Supporting Information

## Data Availability

The data that support the findings of this study are openly available in NCBI at https://doi.org/10.1016/j.cell.2020.03.048, reference number 11.
