# Supplementary material for: NAMPT‐Driven M2 Polarization of Tumor‐Associated Macrophages Leads to an Immunosuppressive Microenvironment in Colorectal Cancer
Source: Adv Sci (Weinh). 2024 Feb 2;11(14):2303177. doi: 10.1002/advs.202303177 (PMC11005718; doi:10.1002/advs.202303177)
Supplement: Supplementary file 1 — Supporting Information [file ADVS-11-2303177-s001.pdf]

ADVANCED SCIENCE
Open Access

Supporting Information

for *Adv. Sci.*, DOI 10.1002/advs.202303177

NAMPT-Driven M2 Polarization of Tumor-Associated Macrophages Leads to an Immunosuppressive Microenvironment in Colorectal Cancer

*Sun Mi Hong, A-Yeon Lee, Byeong-Ju Kim, Jeong-Eun Lee, Su-Yeon Seon, Yu-Jin Ha, Jestlin Tianthing Ng, Gyesoon Yoon, Su Bin Lim, Michael J. Morgan, Jong-Ho Cha, Dakeun Lee and You-Sun Kim**

Supporting Information

NAMPT-Driven M2 Polarization of Tumor-Associated Macrophages Leads to an Immunosuppressive Microenvironment in Colorectal Cancer

Sun Mi Hong[1#], A-Yeon Lee[1,2#], Byeong-Ju Kim[1,2], Jeong-Eun Lee[1,2], Su-Yeon Seon[1,2], Yu-Jin Ha[1,2], Jestlin Tianthing Ng[1,2], Gyesoon Yoon[1,2], Su Bin Lim[1,2], Michael J. Morgan[3], Jong-Ho Cha[4,5], Dakeun Lee[2,6], You-Sun Kim[1,2*]

[1] *Department of Biochemistry, Ajou University School of Medicine, 164 Worldcup-ro, Yeongtong-gu, Suwon, Gyeonggi-do, 16499, Republic of Korea*

[2] *Department of Biomedical Sciences, Graduate School of Ajou University, 164 Worldcup-ro, Yeongtong-gu, Suwon, Gyeonggi-do, 16499, Republic of Korea*

[3] *Department of Natural Sciences, Northeastern State University, Tahlequah, OK, 74464, USA.*

[4] *Department of Biomedical Sciences, College of Medicine, Inha University, Incheon 22212, South Korea.*

[5] *Department of Biomedical Science and Engineering, Graduate School, Inha University, Incheon 22212, South Korea.*

[6] *Department of Pathology, Ajou University School of Medicine, 164 Worldcup-ro, Yeongtong-gu, Suwon, Gyeonggi-do, 16499, Republic of Korea*

[#] These authors contributed equally to this work.

***Corresponding author**

You-Sun Kim, Ph.D.
Department of Biochemistry, Ajou University School of Medicine,
164 Worldcup-ro, Yeongtong-gu, Suwon, Gyeonggi-do, 16499, Republic of Korea
Tel: 82-31-219-4509, Fax: 82-31-219-4530, E-mail: yousunkim@ajou.ac.kr

Fig. S1

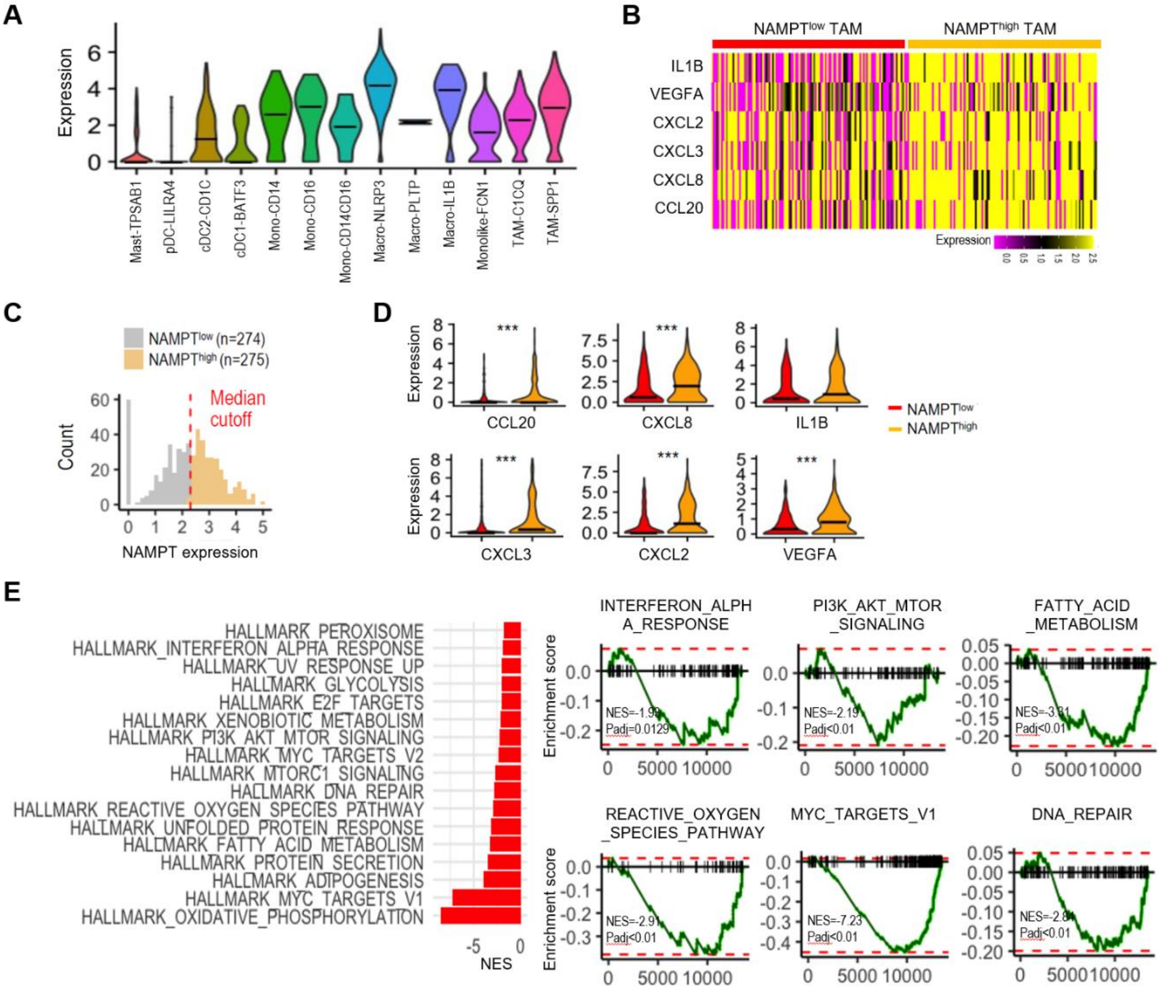
A
Expression
Mast-TPSAB1
pDC-LILRA4
cDC2-CD1C
cDC1-BATF3
Mono-CD14
Mono-CD16
Mono-CD14CD16
Macro-NLRP3
Macro-PLTP
Macro-IL1B
Monolike-FCN1
TAM-C1CQ
TAM-SPP1
B
NAMPTlow TAM
NAMPThigh TAM
IL1B
VEGFA
CXCL2
CXCL3
CXCL8
CCL20
Expression
C
NAMPTlow (n=274)
NAMPThigh (n=275)
Median cutoff
Count
NAMPT expression
D
Expression
CCL20
CXCL8
IL1B
CXCL3
CXCL2
VEGFA
NAMPTlow
NAMPThigh
E
HALLMARK_PEROXISOME
HALLMARK_INTERFERON_ALPHA_RESPONSE
HALLMARK_UV_RESPONSE_UP
HALLMARK_GLYCOLYSIS
HALLMARK_E2F_TARGETS
HALLMARK_XENOBIOTIC_METABOLISM
HALLMARK_PI3K_AKT_MTOR_SIGNALING
HALLMARK_MYC_TARGETS_V2
HALLMARK_MTORC1_SIGNALING
HALLMARK_DNA_REPAIR
HALLMARK_REACTIVE_OXYGEN_SPECIES_PATHWAY
HALLMARK_UNFOLDED_PROTEIN_RESPONSE
HALLMARK_FATTY_ACID_METABOLISM
HALLMARK_PROTEIN_SECRETION
HALLMARK_ADIPOGENESIS
HALLMARK_MYC_TARGETS_V1
HALLMARK_OXIDATIVE_PHOSPHORYLATION
NES
INTERFERON_ALPHA_RESPONSE
PI3K_AKT_MTOR_SIGNALING
FATTY_ACID_METABOLISM
REACTIVE_OXYGEN_SPECIES_PATHWAY
MYC_TARGETS_V1
DNA_REPAIR
Enrichment score
NES=-1.99
Padj=0.0129
NES=-2.19
Padj<0.01
NES=-3.31
Padj<0.01
NES=-2.91
Padj<0.01
NES=-7.23
Padj<0.01
NES=-2.81
Padj<0.01

Supplementary Figure 1. High expression of NAMPT in tumor-specific macrophages, NAMPT^{high}SPP1^{+} TAMs is associated with pro-tumoral TAMs.

A, Relative expression of *NAMPT* in tumor tissues as grouped by myeloid cell clusters. The black line represents median expression.

B, Heatmap showing mRNA expression of *IL1B, VEGFA, CXCL2, CXCL3, CXCL8,* and *CCL*20 in *SPP1*$^{+}$ TAMs, which are clustered into *NAMPT*^{low} and *NAMPT*^{high} groups.

C, Density plot showing distribution of *NAMPT* expression in *NAMPT*^{high} and *NAMPT*^{low} groups in *C1CQ*$^{+}$ TAMs.

D, Relative expression of known TAM markers mediating M1/M2 polarization in *C1CQ*$^{+}$ TAMs. T-test p values (*<0.05, **<0.01, ***<0.001) are shown B. The black line represents median expression.

E, Top enriched Hallmark gene sets in *NAMPT*^{low} group compared to *NAMPT*^{high} group *SPP1*$^{+}$ TAMs. Normalized enrichment scores (NES) are shown (left; adjusted p value<0.05). GSEA plots of selected top-ranked Hallmark gene sets are shown (right). NES and adjusted p values (Padj) are shown.

Fig. S2

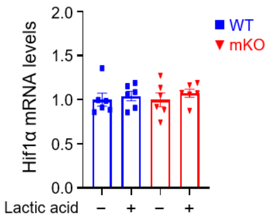

Supplementary Figure 2. NAMPT does not increase the mRNA expression of Hif-1α under lactic acid treatment.

Relative mRNA expression of *Hif-1α* in WT and *Nampt* KO macrophages treated with lactic acid. The mRNA expression of *Hif-1α* was normalized to the mRNA levels of *Tbp1*. Results are represented as the mean ± SEM. Statistical analysis was performed using the unpaired two-tailed Student's *t*-test.

Fig. S3

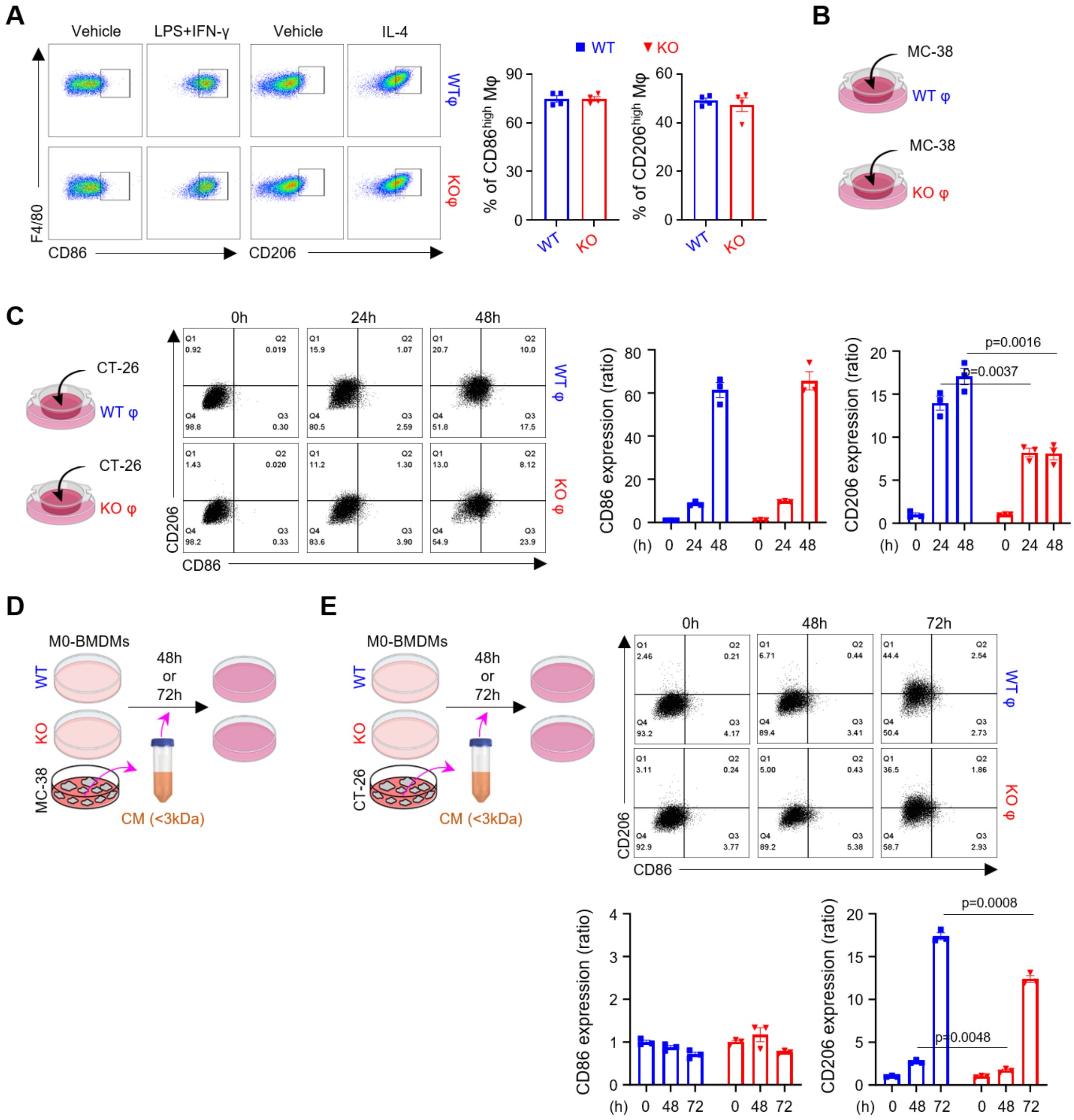

A
Vehicle
LPS+IFN-γ
Vehicle
IL-4
WTφ
KOφ
F4/80
CD86
CD206
■ WT
▼ KO
% of CD86high Mφ
% of CD206high Mφ
WT
KO
B
MC-38
WT φ
MC-38
KO φ
C
CT-26
WT φ
CT-26
KO φ
0h
24h
48h
CD206
CD86
CD86 expression (ratio)
CD206 expression (ratio)
(h) 0 24 48 0 24 48
p=0.0016
p=0.0037
D
M0-BMDMs
WT
KO
MC-38
48h
or
72h
CM (<3kDa)
E
M0-BMDMs
WT
KO
CT-26
48h
or
72h
CM (<3kDa)
0h
48h
72h
WT φ
KO φ
CD206
CD86
CD86 expression (ratio)
CD206 expression (ratio)
(h) 0 48 72 0 48 72
p=0.0008
p=0.0048

Supplementary Figure 3. NAMPT likely drives cell-cell contact-, or secreted factor-mediated M2-like TAM polarization in TME.

A, BMDMs from WT and *Nampt* mKO mice were treated with LPS/IFN-γ or IL-4 and were evaluated for the expression of CD86, CD206 and F4/80 by flow cytometry.

B, Scheme depicting co-culture of BMDMs and colon cancer cells, MC38 cells using transwell plate in figure 3C.

C, BMDMs were co-cultured with CT26 cells using a transwell system for the indicated times. Representative flow cytometry plots and bar graph.

D, Diagram of workflow for figure 3D. The conditioned medium (CM) from MC38 cells was cut off <3kDa using by amicon. BMDMs from WT and *Nampt* mKO mice were cultured in CM (<3kDa).

E, BMDMs from WT and *Nampt* mKO mice were cultured in the CT26 tumor-conditioned medium (<3kDa) for the indicated times (left). Representative flow cytometry plots (middle) and bar graph (bottom). Results are represented as the mean ± SEM. Statistical analysis was performed using the unpaired two-tailed Student's *t*-test.

Fig. S4

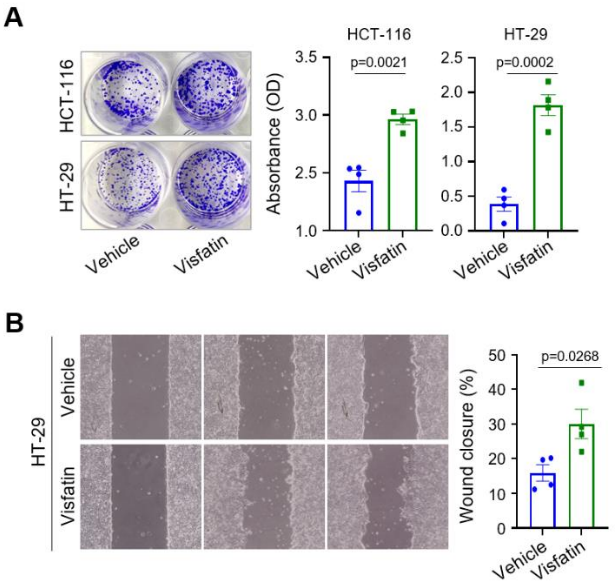

A
HCT-116
HT-29
Vehicle
Visfatin
Absorbance (OD)
p=0.0021
p=0.0002
3.5
3.0
2.5
1.0
2.0
1.5
0.5
0.0
B
Wound closure (%)
p=0.0268
50
40
30
20
10
0

Supplementary Figure 4. Extracellular NAMPT can promote tumor cell malignancy.

A, Colony formation assays for HCT116 cells and HT-29 cells treated with 500 ng/mL extracellular NAMPT (visfatin) for 7 days.

B, Confluent monolayers of HCT116 cells and HT-29 cells were wounded after treatment with 500 ng/mL visfatin and then were cultured for 48 h. Results are represented as the mean ± SEM. Statistical analysis was performed using the unpaired one-tailed Student’s *t*-test.

Fig. S5

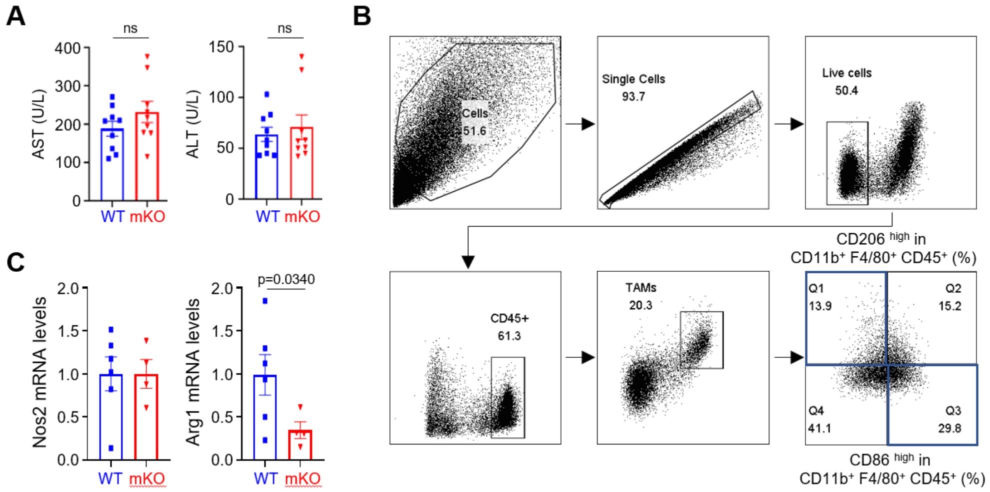
A
ns
400
300
200
100
0
AST (U/L)
WT mKO
ns
150
100
50
0
ALT (U/L)
WT mKO
B
Cells
51.6
Single Cells
93.7
Live cells
50.4
CD45+
61.3
TAMs
20.3
CD206 high in
CD11b+ F4/80+ CD45+ (%)
Q1
13.9
Q2
15.2
Q4
41.1
Q3
29.8
CD86 high in
CD11b+ F4/80+ CD45+ (%)
C
2.0
1.5
1.0
0.5
0.0
Nos2 mRNA levels
WT mKO
p=0.0340
Arg1 mRNA levels
WT mKO

Supplementary Figure 5. NAMPT inhibition reduces tumor outgrowth by limiting the population of immunosuppressive tumor-promoting M2-like TAMs.

A, Serum ALT and AST levels from WT and *Nampt* mKO mice after MC38 cell inoculation.

B, Gating strategy for flow cytometry analysis of tumor tissues from WT and *Nampt* mKO mice is shown. After gating singlet, $CD45^{+}$ cells were analyzed with antibodies of CD11b and F4/80. The flow cytometry gating strategy used to define $CD86^{high}$ $CD206^{low}$ (M1-like) and $CD206^{high}$ $CD86^{low}$ (M2-like) macrophage subpopulation is shown in $CD11b^{+}$ $F4/80^{+}$ macrophages.

C, Relative expression of *Nos2* and *Arg1* mRNA in tumor tissues from WT and *Nampt* mKO mice is shown. The mRNA levels were normalized to the mRNA level of *Tbp1*. Results are represented as the mean ± SEM. Statistical analysis was performed using the unpaired one-tailed Student's *t*-test.

Fig. S6

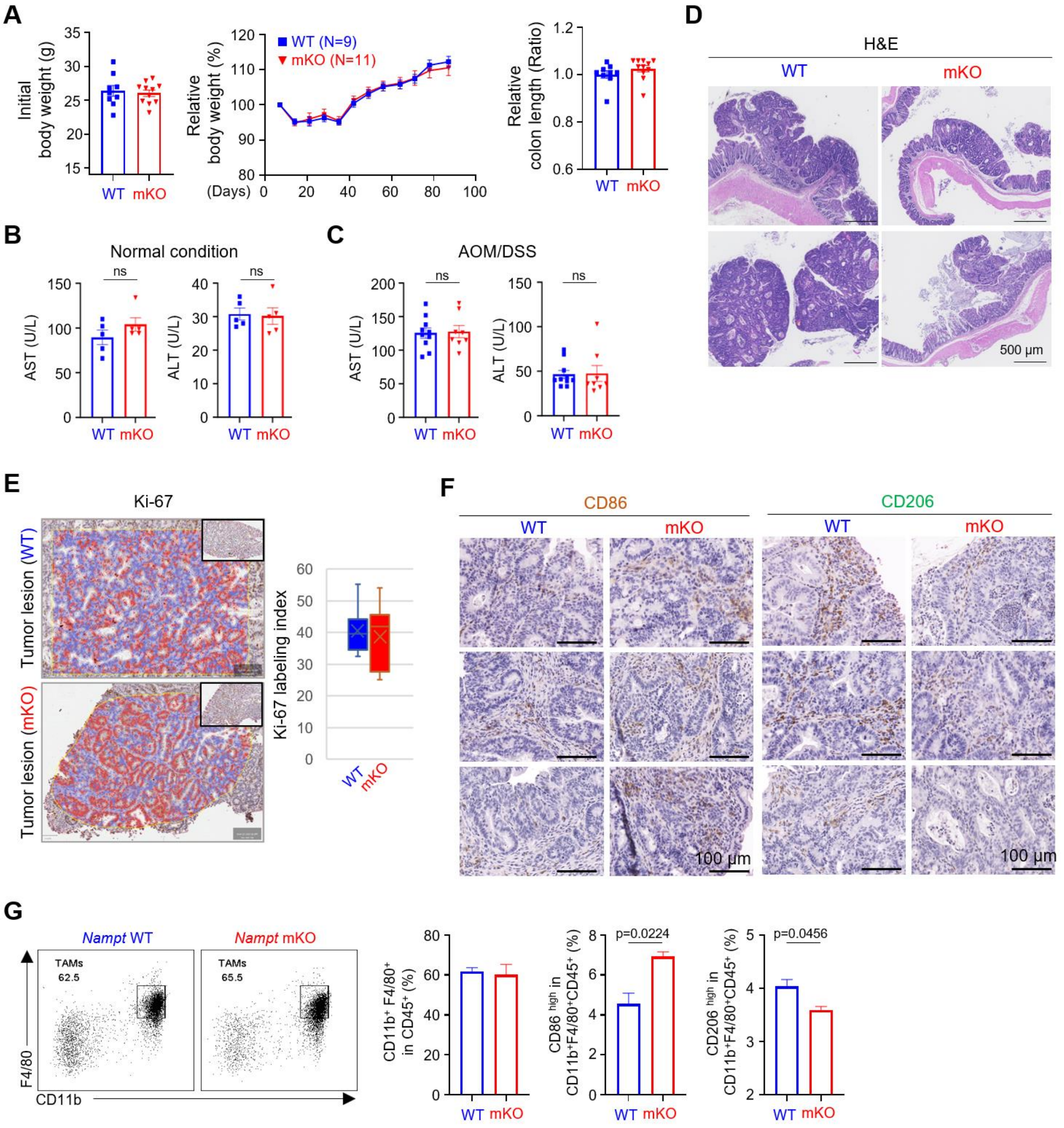

A
Initial body weight (g)
WT mKO
Relative body weight (%)
WT (N=9)
mKO (N=11)
(Days)
Relative colon length (Ratio)
B
Normal condition
ns
AST (U/L)
ALT (U/L)
C
AOM/DSS
D
H&E
500 µm
E
Ki-67
Tumor lesion (WT)
Tumor lesion (mKO)
Ki-67 labeling index
F
CD86
CD206
100 µm
G
Nampt WT
Nampt mKO
TAMs
62.5
65.5
F4/80
CD11b
CD11b+ F4/80+ in CD45+ (%)
CD86 high in CD11b+F4/80+CD45+ (%)
p=0.0224
CD206 high in CD11b+F4/80+CD45+ (%)
p=0.0456

Supplementary Figure 6. FACS analysis shows a reduced M2-like TAMs population in tumor-bearing Nampt mKO mice compared to WT mice.

A, Initial body weight (left), body weight changes (middle) and relative colon length (right) of AOM/DSS-treated mice in figure 5A.

B, Serum ALT and AST levels from WT and *Nampt* mKO mice under normal conditions are shown.

C, Serum ALT and AST levels from WT and *Nampt* mKO mice 8 weeks after AOM/DSS treatment are shown.

D, Representative images of H&E staining of colonic tumor tissues. Scale bar = 500 μm.

E, Quantification of Ki-67 positive areas in colonic tumor tissues from WT and *Nampt* mKO mice treated with AOM/DSS.

F, Immunohistochemistry of CD206 and CD86 in colonic tumor tissues from WT and *Nampt* mKO mice treated with AOM/DSS is shown. Scale bar = 100 μm.

G, Proportion of TAMs among $CD45^+$ immune cells from WT and *Nampt* mKO groups is shown (left). Proportion of $CD86^{high}$ TAMs (M1-like TAMs) and $CD206^{high}$ TAMs (M2-like TAMs) in WT (n=4) and *Nampt* mKO groups (n=2) is shown (right). Results are represented as the mean ± SEM. Statistical analysis was performed using the unpaired one-tailed Student's *t*-test.

Fig. S7

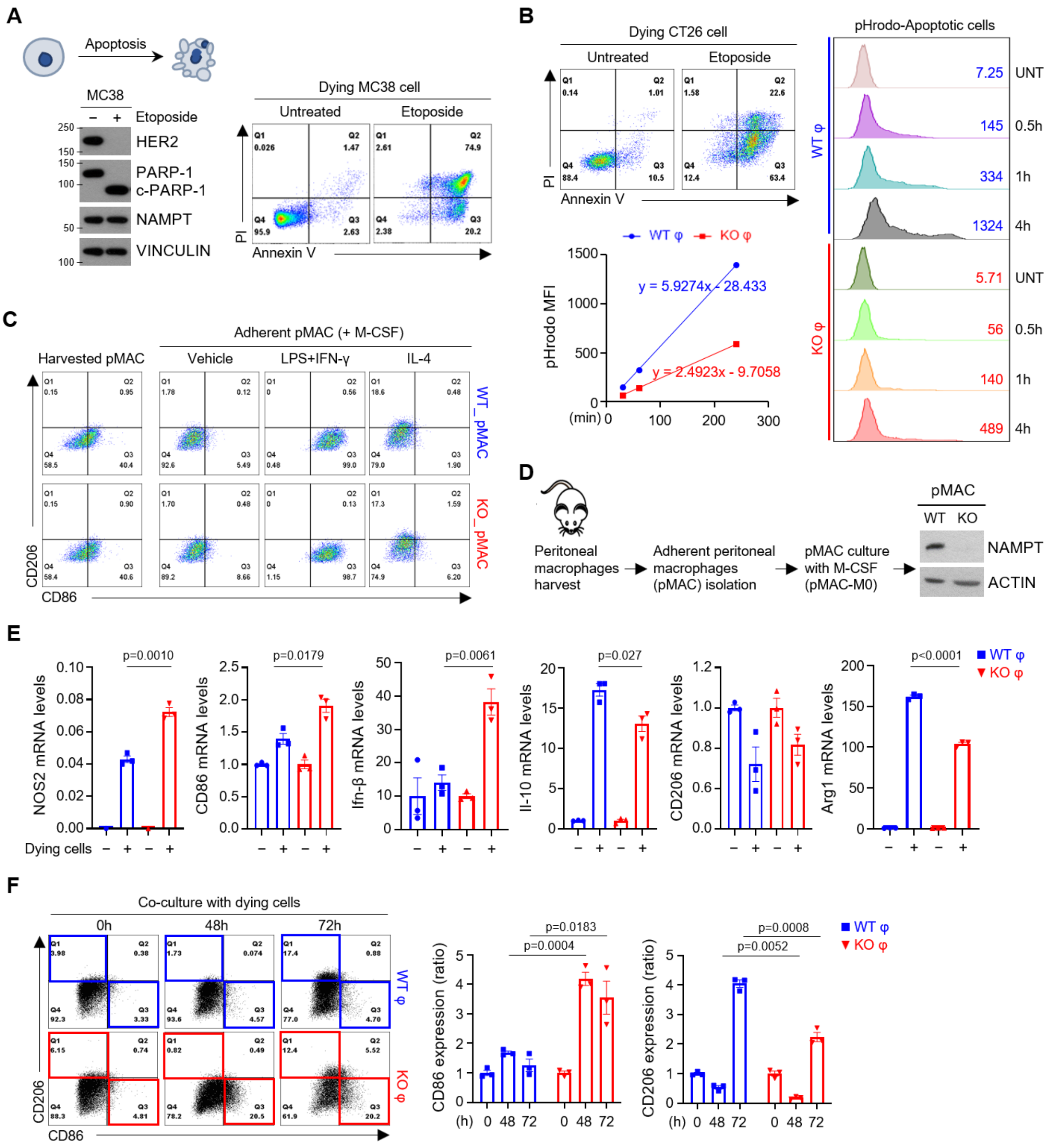

A
Apoptosis
MC38
Etoposide
HER2
PARP-1
c-PARP-1
NAMPT
VINCULIN
Dying MC38 cell
Untreated
Etoposide
PI
Annexin V
B
Dying CT26 cell
Untreated
Etoposide
Annexin V
pHrodo-Apoptotic cells
WT φ
KO φ
UNT
0.5h
1h
4h
pHrodo MFI
y = 5.9274x - 28.433
y = 2.4923x - 9.7058
(min)
C
Adherent pMAC (+ M-CSF)
Harvested pMAC
Vehicle
LPS+IFN-γ
IL-4
WT_pMAC
KO_pMAC
CD206
CD86
D
Peritoneal macrophages harvest
Adherent peritoneal macrophages (pMAC) isolation
pMAC culture with M-CSF (pMAC-M0)
pMAC
WT KO
NAMPT
ACTIN
E
NOS2 mRNA levels
CD86 mRNA levels
Ifn-β mRNA levels
Il-10 mRNA levels
CD206 mRNA levels
Arg1 mRNA levels
Dying cells
p=0.0010
p=0.0179
p=0.0061
p=0.027
p<0.0001
F
Co-culture with dying cells
0h
48h
72h
CD86 expression (ratio)
CD206 expression (ratio)
p=0.0183
p=0.0004
p=0.0008
p=0.0052
(h)

Supplementary Figure 7. NAMPT deficient-macrophages have increased expression of M1 phenotype markers.

A, MC38 cells were treated with 150 μM etoposide for 18 h. Cell lysates were analyzed by western blotting (left). Representative flow cytometry plots using Annexin V/PI staining for apoptosis are shown (right).

B, The phagocytic activity of BMDMs treated with LPS/IFN-γ for 12 h was measured from 30 min to 240 min after adding dying CT26 cells (left upper) labeled with pHrodo green dye using flow cytometry (left bottom). Representative histogram of pHrodo intensity is shown (right).

C, pMAC from WT and *Nampt* mKO mice were treated with LPS/IFN-γ or IL-4 and were evaluated for the expression of CD86 and CD206 in $CD11b^{+}F4/80^{+}$ by FACS.

D, Diagram of workflow for figure 6C. The lysates of pMAC from WT and *Nampt* mKO mice were analyzed by western blotting.

E, BMDMs from WT and *Nampt* mKO mice were directly co-cultured with dying MC38 cells. mRNA expression levels of the indicated genes were analyzed by qRT-PCR. The mRNA levels were normalized to the mRNA level of *Tbp1*.

F, Flow cytometry analysis of $CD86^{high}$ and $CD206^{high}$ populations in $CD11b^{+}F4/80^{+}$ BMDMs co-cultured with dying MC38 cells is shown. Representative flow cytometry plots (left) and bar graph (right). Results are represented as the mean ± SEM. Statistical analysis was performed using the unpaired two-tailed Student's *t*-test.

Fig. S8

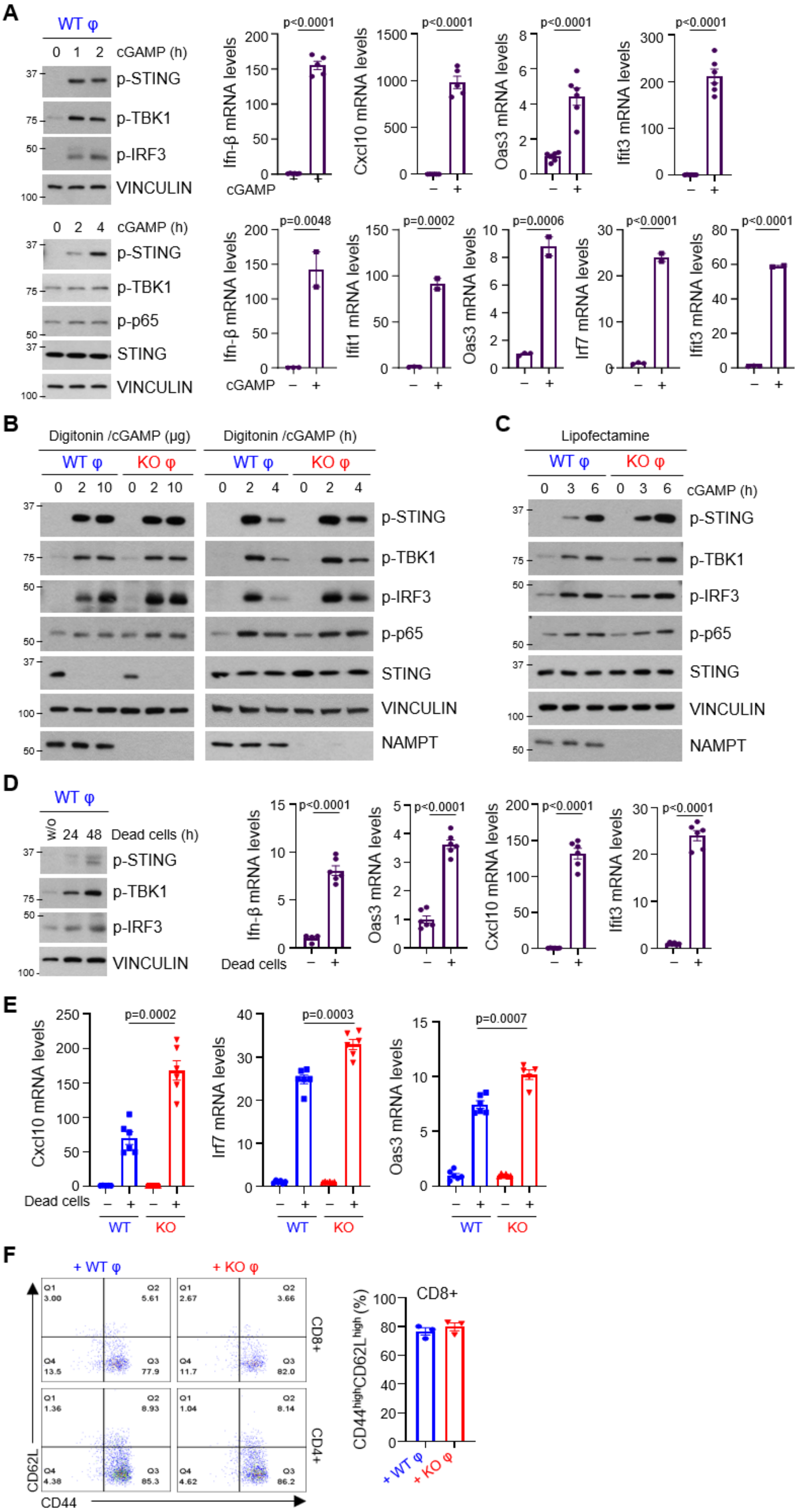
A
WT φ
cGAMP (h)
p-STING
p-TBK1
p-IRF3
VINCULIN
p-p65
STING
Ifn-β mRNA levels
Cxcl10 mRNA levels
Oas3 mRNA levels
Ifit3 mRNA levels
Ifit1 mRNA levels
Irf7 mRNA levels
p<0.0001
p=0.0048
p=0.0002
p=0.0006
B
Digitonin /cGAMP (μg)
Digitonin /cGAMP (h)
WT φ
KO φ
NAMPT
C
Lipofectamine
D
Dead cells (h)
Dead cells
E
p=0.0002
p=0.0003
p=0.0007
WT
KO
F
+ WT φ
+ KO φ
CD8+
CD4+
CD62L
CD44
CD44^high CD62L^high (%)

Supplementary Figure 8. NAMPT deficiency in macrophages promotes anti-tumoral immunity through STING-dependent type I IFN responses.

A, WT BMDMs were treated with 10 μg cGAMP by using 5 μg/mL digitonin permeabilization buffer (upper panel) or lipofectamine (lower panel) for the indicated times. Cell lysates were analyzed by western blotting (left). mRNA levels of *Ifn-β* and IFN response genes were analyzed by qRT-PCR (right). mRNA levels of the indicated genes were normalized to the mRNA level of *Tbp1*.

B-C, BMDMs from WT and *Nampt* mKO mice were treated with cGAMP by using 5 μg/mL digitonin permeabilization buffer (B) or lipofectamine (C). Cell lysates were analyzed by western blotting using the indicated antibodies.

D, WT BMDMs were treated with dying MC38 cells for the indicated times and cell lysates were analyzed by western blotting (left). mRNA levels of *Ifn-β* and IFN response genes were analyzed by qRT-PCR (right). mRNA levels of the indicated genes were normalized to the mRNA level of *Tbp1*.

E, BMDMs from WT and *Nampt* mKO mice were incubated with dying MC38 cells for 24 h. The mRNA expression of IFN response genes were analyzed by qRT-PCR. mRNA expression of the indicated genes was normalized to the mRNA level of *Tbp1*.

F, M0 BMDMs from WT and *Nampt* mKO mice were incubated with splenocytes from naïve mice for 2 days. FACS analysis of the proportion of effector cells ($CD44^{high}CD62L^{low}$) of $CD8^+$ T cells co-cultured with TAMs is shown. Representative flow cytometry plots (left) and bar graph (right). Results are represented as the mean ± SEM. Statistical analysis was performed using the unpaired two-tailed Student's *t*-test.

Supplementary Table

Table S1. Primer sequences used in qRT-PCR

Species	Gene name		Sequence
mouse	*Il-6*	F	AGTTCCTCTCTGCAAGAGACT
		R	ATGTGTAATTAAGCCTCCGACTT
	Il-10	F	TTACTGACTGGCATGAGGATCA
		R	AAGGAGTCGGTTAGCAGTATGT
	Tgf-β	F	AGGGCTACCATGCCAACTTC
		R	CCACGTAGTAGACGATGGGC
	Tnf-α	F	TATGTCTCAGCCTCTTCTCATTC
		R	GTCACTCGAATTTTGAGAAGATGAT

	Il-17	F	GCTGACCCCTAAGAAACCCC
		R	GAAGCAGTTTGGGACCCCTT
	Ifn-β	F	AACTATAAGCAGCTCCAGCTC
		R	CTTGGATGGCAAAGGCAGTG
	Cd206	F	ATCTCTGTCATCCCTGTCTCT
		R	ACTCATGATCTGAGAATCTGACA
	Arg-1	F	AAGAATGGAAGAGTCAGTGTGGT
		R	TGCAGATTCCCAGAGCTGGT
	Spp1	F	TGACCCATCTCAGAAGCAGA
		R	TGGTCTCCATCGTCATCATCAT
	Pdgf	F	CACAGAGACTCCGTAGATGAAG
		R	TTGCACTCGGCGATTACAG
	Flt1	F	GATATGGCTCAGGGTCGAA
		R	GTGCTGCAGAATTGCCTGTTATC
	Mct1	F	TGTTAGTCGGAGCCTTCATTTC
		R	CACTGGTCGTTGCACTGAATA
	Mct4	F	TCAATCATGGTGCTGGGA
		R	GGCAAAGCTGAAGAGGTAGA
	Tyro3	F	AACGCATTGAGGCCACATT
		R	CTTGAGGCAATGATGTCAGCTTT
	Axl	F	GAAAGAGGTGAACTGGTAGTCA
		R	ATTGAGCTGACCTTCCATCAC
	Mertk	F	CTGTAGCACCTTTAAACATCACTGTG
		R	CATTGTGGATTTGGACAGGAATCT
	Ifit3	F	AATCTCTGGAAGCGATCCTTC
		R	ACATTGTTGCCTTCTCCTCAGA

mouse	*Oasl1*	F	AGAGTGGAAAGAAGAGGTCCT
		R	TGTGGAAACAGCTCAGGAACA
	Ccl5	F	TCACCATCATCCTCACTGCA
		R	AAGATTGGAGCACTTGCTGCT
	Cd86	F	ATGCACCATGGGCTTGGCAA
		R	AACTTTTGCTGGTCCTGCCAAA
	Nos2	F	TCTTCAAAGTCAAATCCTACCAAAG
		R	TCGATGTCACATGCAGCTTGT
	Ifit1	F	TCAGCTGAGATGTCACTTCACA
		R	AGCTGCTCGCTCTGGATCAA
	Cxcl10	F	CATTTTCTGCCTCATCCTGCT
		R	TGGTCTTAGATTCCGGATTCAG

	Oas3	F	CAGAGACCAAGAGTGATAAGGA
		R	ATCCTGGACGCCTTTGACTG
	Irf7	F	TGAACGAGGCTCGCACAGT
		R	TCTTCCAGCCTCTTCGCTCT
